# Reliability of bedside tests for diagnosing peripheral arterial disease in patients prone to medial arterial calcification: A systematic review

**DOI:** 10.1016/j.eclinm.2022.101532

**Published:** 2022-07-01

**Authors:** Jeroen J.W.M. Brouwers, Siem A. Willems, Lauren N. Goncalves, Jaap F. Hamming, Abbey Schepers

**Affiliations:** a Department of Vascular Surgery, Leiden University Medical Center, Leiden, the Netherlands; b Department of Surgery, Haga Teaching Hospital, the Hague, the Netherlands; c Department of Surgery, Haaglanden Medical Center, the Hague, the Netherlands

**Keywords:** Peripheral arterial disease, Diagnosis, Non-invasive diagnostics, Medial arterial calcification, Diabetes mellitus, Chronic kidney disease, Systematic review

## Abstract

**Background:**

Medial arterial calcification (MAC), frequently associated with diabetes mellitus (DM) and chronic kidney disease (CKD), is a systemic vascular disorder leading to stiffness and incompressible arteries. These changes impede the accuracy of bedside tests to diagnose peripheral arterial disease (PAD). This review aimed to evaluate the reliability of bedside tests for the detection of PAD in patients prone to MAC.

**Methods:**

A systematic search (Pubmed, Embase, Web of Science, Cochrane, and Emcare) was performed according to the PRISMA guidelines to identify relevant studies providing data on the performance of bedside tests for the detection of PAD in patients prone to MAC. Studies were included when bedside test were compared to a reference standard. Primary endpoints were the positive and negative likelihood ratios (PLR, NLR). Methodological quality and risk of bias were evaluated using the QUADAS-2 tool.

**Findings:**

In total, 23 studies were included in this review. The most commonly evaluated test was the ankle-brachial index (ABI), followed by toe-brachial index (TBI), toe pressure (TP) measurements, and continuous wave Doppler (CWD). The majority of patients were older, male, and had DM. We found that ABI <0·9 was helpful to diagnose PAD, but failed to rule out PAD (NLR >0·2). The same applied for TP (NLR >0·3) and TBI (5 out of 6 studies revealed an NLR >0·2). CWD (loss of triphasic pattern) is reliable to exclude PAD (NLR 0-0·09), but was only validated in two studies. Overall, methodological quality was poor which led to risk of bias in 20 studies.

**Interpretation:**

The diagnosis of PAD in patients prone to MAC remains challenging. The ABI performed reasonably in the diagnosis of PAD, while the CWD (loss of triphasic signal) can be used to rule out PAD. This systematic review showed that test performances were generally poor with serious concerns in methodological quality of the included studies. We therefore counsel against the use of a single bedside test.

**Funding:**

None to declare.

**Research in context**

**Evidence before this study**

Peripheral arterial disease (PAD) is an increasing problem worldwide and is intertwined throughout all medical care. Medial arterial calcification (MAC), common in diabetes mellitus and chronic kidney disease, decreases the accuracy of bedside tests leading to a challenge in daily clinical practice. Early identification of PAD is particularly needed in these patients, allowing for the prompt initiation of cardiovascular risk management (CVRM) and thus reduce the risk of events.

**Added value of this study**

This systematic review compiled 23 diagnostic studies regarding 5404 patients prone to MAC. Overall, no singular bedside test showed sufficient ability to diagnose and rule out PAD in this patient group. The ankle-brachial index (<0.9 and exclusion of >1.3) seemed useful to diagnose PAD, while the continuous wave Doppler (loss of triphasic signal) provided reliable performance to rule out PAD.

**Implications of all the available evidence**

Both for ruling in and ruling out PAD, the performance of current bedside tests was disappointing. Generally, risk of bias was high in the included studies with respect to patient selection and interpretation of the bedside tests. These results should strengthen guideline recommendations to renounce the use of a singular bedside test for patients prone to MAC.

Alt-text: Unlabelled box

## Introduction

Peripheral arterial disease (PAD) of the lower extremity is considered a clinical manifestation of systemic atherosclerosis. It is estimated that more than 200 million people are suffering from PAD worldwide.^1^ Non-invasive bedside tests such as the ankle-brachial index (ABI) are considered accurate for the diagnosis of PAD. However, the accuracy of bedside testing can be affected by medial arterial calcification (MAC), leading to falsely elevated and unreliable results.^2^, ^3^, ^4^, ^5^, ^6^

MAC is a complex and poorly understood pathological process resulting in incompressible arteries due to calcification of the media of the arterial wall. The increase in arterial wall stiffness impedes bedside diagnostic tools reliant on hemodynamic changes to detect PAD.^7^^,^^8^ This process is thought to be characteristic of aging, and is expedited in the presence of diabetes mellitus (DM) and chronic kidney disease (CKD).^9^, ^10^, ^11^ Research suggests that MAC is present in approximately one third of patients with DM, and up to 70% in amputations for critical limb ischemia.^12^, ^13^, ^14^ MAC has been shown to be an independent predictor of cardiovascular mortality, while another study found that patients with DM and PAD have an impaired quality of life and an increased risk of adverse cardiac and limb events.^15^^,^^16^

While the accurate diagnosis of PAD in patients with MAC can be challenging, timely recognition of critical limb ischemia and initiation of treatment in this patient population is pertinent to reduce delayed wound healing, prevent (major) lower limb amputation, and mortality in diabetic patients with PAD.^17^^,^^18^ It is expected that the number of patients with DM will increase to nearly 370 million people by 2030 worldwide.^19^ Therefore, reliable non-invasive bedside tests to diagnose PAD in patients prone to MAC is of the utmost importance. Recently two systematic reviews were published regarding bedside tests in patients with DM.^20^^,^^21^ However, bedside diagnostics should be tested in a wider context. MAC causes incompressible arteries and is the underlying problem of the poor performance of the bedside tests. Thus, bedside tests must not only be investigated in patients with DM, but in all patients prone to MAC such as patients with CKD and an ABI >1.3. A complete overview of the diagnostic performance of bedside tests in patients prone to MAC is lacking. Therefore, the aim of this systematic review is to evaluate the reliability of bedside tests compared to reference imaging techniques for diagnosing PAD in patients prone to MAC.

## Methods

### Search strategy

This study was conducted according to the PRISMA (Preferred Reporting Items for Systematic reviews and Meta-Analyses) guidelines^22^ and was not registered in a registry. A literature search was performed in PubMed, Embase (OVID-version), Web of Science, Cochrane Library, and Emcare until February 2021. The search string and justification of the strategy can be found in Supplement S1. Two reviewers (JB, SW) independently screened the titles and abstracts for eligibility of inclusion. Disagreements were resolved in a discussion meeting between two reviewers (JB, SW). Full text articles of the selected abstracts were assessed for inclusion, and the data was extracted.

### Selection criteria

We aimed to evaluate the reliability of bedside tests compared to reference tests to diagnose PAD. Bedside tests were considered as any non-invasive technique to detect PAD at the point-of-care. These tests should also be readily available and easy in use. To be eligible for inclusion, studies were required to comply with the following criteria: I) evaluated a bedside (e.g. ABI, TBI, toe pressure, oximetry, pulsations, Doppler waveform) index test compared to a reference test; II) All included patients in the (sub)analyses had to be prone to MAC, defined as DM, CKD or ABI >1·3; III) published in English.

Although digital subtraction angiography (DSA) is regarded as the gold standard for the diagnosis of PAD, it is invasive and carries risks. Magnetic resonance angiography (MRA),^23^ computed tomography angiography (CTA),^24^ and duplex ultrasonography (DUS)^25^ have all been proven to accurately diagnose PAD, and were thus included as reference tests as well. The primary outcomes of interest regarding diagnostic accuracy were the positive likelihood ratio (PLR) and negative likelihood ratio (NLR), because these outcomes reflect the test's ability to rule in or rule out disease (PAD). The interpretation of these likelihood ratios is shown in Table 1. Furthermore, sensitivity and specificity of the index tests were also mentioned. We excluded articles that compared bedside tests to each other, reported insufficient data about PLR, NLR, sensitivity, and specificity, investigated serum markers, or were case reports.

**Table 1:** The interpretation of likelihood ratios and their effect on post-test probability of disease.^51^

Positive likelihood ratio (PLR)	Negative likelihood ratio (NLR)	Interpretation: effect on ability to rule in/rule out disease
>10	<0·1	Large
5-10	0·1-0·2	Moderate
2-5	0·2-0·5	Small
1	1	No change

### Data extraction and quality assessment

Data extraction was performed and verified independently by two investigators (JB, SW). For all articles, extracted data consisted of relevant patient characteristics, the index test performed, correlated imaging modalities, and the diagnostic value (PLR, NLR, sensitivity and specificity) of the index test compared to a reference standard. Measures of test performance such as PLR, NLR, sensitivity, and specificity were extracted and calculated (if necessary) from the accessible data.

Methodological quality and risk of bias were assessed using the Quality Assessment of Diagnostic Accuracy Studies-2 (QUADAS-2) tool, specifically designed for diagnostic accuracy studies.^26^ Due to heterogeneity in patient selection, clinical diversity, and the threshold values of index- and reference tests, a meta-analysis could not be performed.

### Role of the funding source

There is no direct or indirect funding to declare. Authors JB and SW had access to the data and took the decision to submit for publication.

## Results

### Overview of studies

**Figure 1:**
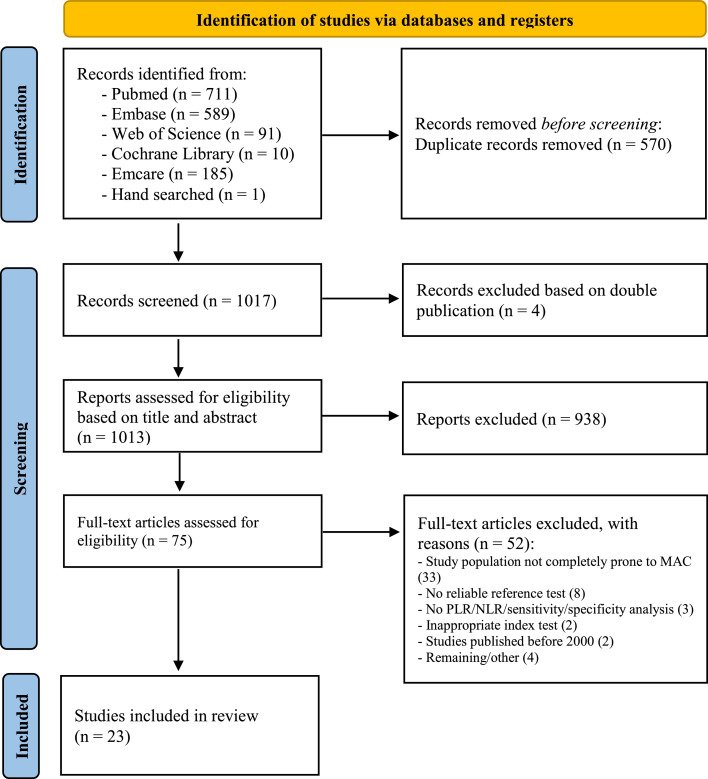
Flow diagram illustrating article selection process according to the PRISMA guidelines.^22^

An overview of the article selection for this systematic review is reported according to the PRISMA 2020 guidelines (Figure 1).^22^ A total of 1017 articles were found, of which 23 studies were eventually included, comprising of 6869 patients. Thirteen of the 23 selected studies included solely patients prone to MAC, described as DM, CKD, or incompressible arteries (n=4038). A sub-analysis specifically assessing test performance in patients prone to MAC was performed in the other ten studies (n=1366). Of the studies selected, 12 were prospective cohort or cross-sectional studies (n=3847), nine were retrospective studies (n=2837), and two were prospective case-control studies (n=185). In the 23 included studies, the number of study participants ranged from 16^27^ to 2188,^28^ and the ages of subjects at baseline ranged from 53 to 77 years old. The diagnosis of DM was specifically noted in 3693 patients. Duration of DM was mentioned in 12 studies,^28^, ^29^, ^30^, ^31^, ^32^, ^33^, ^34^, ^35^, ^36^, ^37^, ^38^ and ranged from 2 to 24 years. Eleven of the included studies described the application of multiple bedside tests per patient population, while twelve studies explored the diagnostic value of a singular bedside test, as shown in Table 2. The ABI was the most commonly evaluated bedside test, mentioned in 18 of the 23 included studies. Table 2 describes the 13 other diagnostic parameters discussed in this review. Seventeen studies used DUS as the reference standard for confirming the presence of PAD, and mostly defined >50% stenosis as cut-off value (12 studies). Alternative reference tests included MRA in three studies, CTA in one study, and DSA in two studies.

**Table 2:** Evidence table of all included studies.

Author & year (ref)	Country	Study design & setting	Population (n, age, gender, comorbidity, patient characteristics)	Index/non-invasive/point of care test	Reference test; definition of PAD	Index test performance (sensitivity/specificity/PLR/NLR)			Comments/opinion
AbuRahma et al.^40^ 2020	United States of America	Single-center retrospective cohort study	**Overall** N = 1162 patients with symptomatic PAD Mean age: 65·4 years Gender: not specified 57% of patients had claudication symptoms 43% of patients had limb threatening ischemia **Subgroup analysis** Diabetes (46%: 535 patients) CKD (16%: 186 patients) Age/gender: not specified	ABI <0.9 TBI <0·7	DUS (PAD was defined as >50% stenosis)	*Current results only include the subgroup analysis.* **ABI:** **Diabetics** Sens: 51 (46·1-56·3) Spec: 89 (84·3-92·5) PLR: 4·64 NLR: 0·55 **TBI:** **Diabetics** Sens: 84 (76·0-90·3) Spec: 58 (46·1-69·9) PLR: 2·0 NLR: 0·28		**CKD** Sens: 43 (34·3-52·7) Spec: 95 (88·7-98·4) PLR: 8·6 NLR: 0·6 **CKD** Sens: 77 (61·4-88·2) Spec: 64 (42·5-82·0) PLR: 2·14 NLR: 0·36	The proportion of patients who had TBI is unclear. The proportion of patients who had a reference test is unclear in the specific subgroups.
*Aubert et al.^29^ 2014	France	Single-centercross-sectional cohort study	N = 200 patients with diabetes (400 lower limbs) Mean age: 65 years Gender: 80% male Mean duration of DM was 13 years	ABI ≤0·90 or ≥1·30 Foot pulses missing or weak	DUS (PAD was defined as >70% stenosis)	**ABI:** Sens: 42·3% Spec: 80% PLR: 2·11 NLR: 0·72 **Foot pulses missing or weak:** Sens: 69·2% Spec: 71·9% PLR: 2·46 NLR: 0·43			Patients with CKD (eGFR < 30 ml/min) were excluded.
Buschmann et al.^41^ 2018	Austria	Single-centerprospective cohort study	**Overall** N = 166 patients suspected of PAD Mean age: 70 years Gender: 76% male **Subgroup analysis** Diabetes (76 patients) Mean age: 70 years Gender: 68% male Hypertension: 89% CVD: 82% CKD: 25%	ABI ≤0·88 ACC_max_ <4·4 m/sec^2^ Relative Pulse Slope Index (RPSI) 58·00s^−1^	DSA (PAD was defined as >50% stenosis)	*Current results only include the subgroup analysis.* **ABI:** Sens: 56% Spec: 83% PLR: 3·29 NLR: 0·53 **ACCmax:** Sens: 57% Spec: 98% PLR: 28·5 NLR: 0·44		**RPSI:** Sens: 57% Spec: 95% PLR: 11·4 NLR: 0·45	Only patients with ABI of ≤0·90 or ≥1·30 and >25% stenosis at DUS were referred for DSA. The proportion is unclear. Patients who were diagnosed with atherosclerotic stenosis of >25% but who did not have a DSA available were excluded. The proportion is unclear.
Clairotte et al.^42^ 2009	France	Single-centerprospective cohort study	**Overall** N = 146 consecutive patients (292 lower limbs), referred to the physiology department for Doppler ultrasound evaluation of PAD Mean age: 62 years Gender: 68% male **Subgroup analysis** Diabetes (83 patients) Mean age: 63 years Gender: 61% male	Doppler and oscillometric derived ABI <0·90	DUS (PAD was defined as systolic velocity ratio >2·0)	*Current results only include the subgroup analysis.* **Doppler ABI:** Sens: 54% Spec: 97% PLR: 17 NLR: 0·47 **Oscillometric ABI:** Sens: 29% Spec: 96% PLR: 7·9 NLR: 0·74			Unblinded study The NLR was recalculated by the present research group since the NLR results in the original paper were incorrect.
Faglia Ezio et al.^30^ 2010	Italy	Single-centerprospective cohort study	N = 261 patients with diabetes and rest pain and/or foot ulcer in 1 limb Mean age: 73 years Gender: 67% male Mean duration of DM was 18 years	Ankle pressure (AP) <70 mm Hg Transcutaneous oxygen tension (TcPO_2_) <50 mm Hg	DSA (PAD was defined as >50% stenosis)	**AP:** Sensitivity: 33% Spec: N/A PLR: N/A NLR: N/A **TcPO_2_ ≤30 mmHg:** Sensitivity: 82% Spec: N/A PLR: N/A NLR: N/A **TcPO_2_ ≤50 mmHg:** Sensitivity: 100% Spec: N/A PLR: N/A NLR: N/A			Unblinded study Only patients with AP <70 mm Hg and/or TcPO_2_ <50 mm Hg underwent DSA. All included patients had >50% stenosis on DSA probably due to the selection of patients. As a result, it was not possible to calculate specificity. AP not measurable in 42% patients (13% arterial calcifications).
Homza et al.^31^ 2019	Czech Republic	Single-centerprospective cohort study	N = 62 patients with diabetes (124 limbs) Mean age: 68 years Gender: 74% male Mean duration of DM was 8 years	Doppler ABI using highest ankle pressure (hABI) <0·9 or >1·4 Doppler ABI using lowest ankle pressure (lABI) <0·9 or >1·4 Oscillometric ABI <0·9 or >1·4	DUS (PAD was defined as >50% stenosis)	**Higher ABI:** Sens: 67% Spec: 75% PLR: 2·68 NLR: 0·44 **Oscillometric ABI:** Sens: 61% Spec: 94% PLR: 10·17 NLR: 0·41		**Lower ABI:** Sens: 87% Spec: 76% PLR: 3·63 NLR: 0·17	Patients with critical limb ischemia were excluded (Rutherford 4-6).
Hur et al.^32^ 2018	South Korea	Single-centerretrospective cohort study	N = 324 patients with diabetes Mean age: 63 years Gender: 59% male Mean duration of DM was 11 years	ABI <0·9	DUS (PAD was defined as >50% stenosis)	**ABI:** Sens: 17% Spec: 99% PLR: 17 NLR: 0·84			Patients with ABI >1·40 were excluded.
Janssen et al.^33^ 2005	Germany	Single-centerprospective cohortstudy	N = 106 patients with diabetes who were hospitalized Mean age: 72 years Gender: 68% male Mean duration of DM was 20 years	ABI <0·9 Ankle-Brachial Pressure (ABP) <70 mmHg Pulsatility index (PI) <1·2	The need for revascularization on the basis of a) clinical findings and b) arteriographic findings.	**ABI:** Sens: 71% Spec: 42% PLR: 1·22 NLR: 0·69 **ABP:** Sens: 30% Spec: 89% PLR: 2·73 NLR: 0·79		**PI:** Sens: 87% Spec: 62% PLR: 2·29 NLR: 0·21	In total, 54% of patients had medial arterial calcification (assessment on X-ray).
Li et al.^28^ 2015	China	Single-centercross-sectional cohort study	**Overall** N = 2188 patients with diabetes Mean age: 61 years Gender: 54% male **Subgroup analysis:** ABI > 1·3 (175 patients) Mean age: 63 years Gender: 59% male Mean duration of DM was 9 years	ABI >1·45	DUS and MRA 438 underwent DUS/MRA due to abnormal ABI: - 314 patients had DUS - 124 patients had MRA	*Current results only include the subgroup analysis.* **ABI ≥1·45:** Sens: 65% Spec: 85% PLR: 4·33 NLR: 0·41			The optimal ABI threshold was calculated (determined with Youden index). Threshold of reference test to diagnose PAD was unclear.
*Normahani et al.^39^ 2020	United Kingdom	Multicenter prospective cohort study	N = 305 patients with diabetes (recruited from diabetic foot clinics) Mean age: 72 years Gender: 68% male Mean duration of DM was 17 years CKD was present in 17% of patients	Pulse palpation (absence of dorsalis pedis or posterior tibial artery pulse) Audible Doppler (monophasic or absent signal in either vessel) Visual Doppler with handheld Doppler device (monophasic or absent signal in either vessel) ABI < 0.9 TBI < 0.75 TcPO2 <40 mmHg PAD-scan (the presence of an occlusion, venous like slow flow, monophasic waveform or a biphasic waveform with adverse features in either vessel)	DUS (PAD was defined as >50% stenosis)	**ABI** Sens: 60% Spec: 75% PLR: 2.46 NLR: 0.53 **Audible Doppler** Sens: 74% Spec: 76% PLR: 3.04 NLR: 0.35 **Visual Doppler** Sens: 83% Spec: 75% PLR: 3.28 NLR: 0.23 **Pulse palpation** Sens: 43% Spec: 81% PLR: 2.22 NLR: 0.71	**TBI** Sens: 60% Spec: 86% PLR: 4.26 NLR: 0.47 **TcPO2** Sens: 31% Spec: 79% PLR: 1.43 NLR: 0.88 **PAD-scan** Sens: 95% Spec: 77% PLR: 4.06 NLR: 0.07		PAD-scan was performed using a portable ultrasound machine with a linear 6-14Hz transducer. A ‘normal’ biphasic waveform indicated no PAD. However, several adverse features are mentioned in this study leading biphasic waveforms to abnormal: - Spectral broadening - Infilling of the spectral window - Long diastolic forward flow - Slow systolic rise time
Perriss et al.^43^ 2005	Denmark	Single-centerretrospective cohort study	N = 104 patients with end-stage renal failure who underwent CE-MRA of the lower extremity Mean age: 53 years Gender: 71% male 80 asymptomatic patients 24 symptomatic patients (16 claudication, 5 ulcers, 3 other symptoms) **Study population consisted of 69 patients (had both ABI and MRA)**	ABI <0·90	CE-MRA (PAD was defined as >50% stenosis)	**ABI in asymptomatic patients (n=48):** Sens: 56·3% Spec: 87·5% PLR: 4·50 NLR: 0·50 **ABI in combined patients (n=69):** Sens: 74·3% Spec: 85·3% PLR: 5·05 NLR: 0·30		**ABI in symptomatic patients (n=21):** Sens: 89·5% Spec: 50% PLR: 1·79 NLR: 0·21	In 80 out of 104 patients, the indication for MRA was pretransplant evaluation (asymptomatic). 19 of 80 asymptomatic patients had incompressible vessels (24%).
Premalatha et al.^34^ 2002	India	Single-centerprospective cohort study	N = 100 hospital admitted patients with diabetes and severe foot infections Mean age: 60 years Gender: not specified Mean duration of DM was 12 years	ABI <0·90	DUS (PAD was defined as >50% stenosis)	**ABI:** Sens: 70·6% Spec: 88·5% PLR: 6·14 NLR: 0·33			Six patients with calcification of peripheral vessels were excluded (unclear how presence of calcification was assessed).
Ro et al.^44^ 2013	South Korea	Single-centerretrospective cohort study	N = 97 patients (194 legs), who had coincidentally undergone CTA, PPG, ABI and CWD for the evaluation of PAD Mean age: 67 years Gender: 91% male **Subgroup analysis** Diabetes (44 patients, 88 legs) Mean age/gender: not specified	ABI <0.90 Continuous-Wave Doppler (CWD), considered positive if: (1) Loss of triphasic pattern, or (2) Decreased amplitude of more than 50% compared with the contralateral side, or (3) Loss of reverse flow component. Photoplethysmography (PPG) wave form, considered positive if: (1) Loss of dicrotic Notch, or (2) Decreased amplitude of more than 50% compared with contralateral side, or (3) rounding of peaks compared with contralateral side.	CTA (PAD was defined as >50% stenosis)	*Current results only include the subgroup analysis.* **ABI:** Sens: 74·7(64-83) Spec: 88.9(57-98) PLR: 6·73 NLR: 0·28 **PPG:** Sens: 78·5 (68-86) Spec: 89% (57-98) PLR: 7·14 NLR: 0·24		**CWD:** Sens: 97·5 (91-99) Spec: 66·7 (35-88) PLR: 2·93 NLR: 0·04	
Saunders et al.^27^ 2019	United Kingdom	Single-centerretrospective cohort study	N = 16 patients (32 limbs) Mean age: 66 years Gender: 94% male Selection criteria included confirmed incompressible vessels (defined as persistent flow with blood pressure cuff inflated to >220 mm Hg) and MRA within the preceding 6 months with no interval arterial intervention. All patients had tissue loss	Vascular early warning system (VEWS) device	MRA (PAD was defined as >50% stenosis)	**VEWS ≤0·94:** Sens: 73% Spec: 80% PLR: 3·65 NLR: 0·34			VEWS functions by using red and infrared optical sensors placed on the toe and dorsum of the foot to register changes in blood volume within the microvasculature that occur during a gravity-induced functional test.
Sonter et al.^47^ 2017	Australia	Single-centerprospective cohort study	**Overall** N = 90 patients (PAD analysis) Mean age: 73 years Gender: 58% male **Subgroup analysis** Diabetes (50 patients) Mean age/gender: not specified	TBI <0·70 Toe pressure <70 mmHg	DUS (PAD was defined as >50% stenosis)	*Current results only include the subgroup analysis.* **TBI:** Sens: 73·9% Spec: 66·7% PLR: 2·22 NLR: 0·39 **Toe pressure:** Sens: 45·8% Spec: 100% PLR: infinite NLR: 0·54			32% of patients had medial arterial calcification. However, it was unclear how presence of medial arterial calcification was assessed. It was unclear if TBP <70 was pre-specified.
Tehan et al.^45^ 2016	Australia	Single-center prospective cross- sectional case-control study	**Overall** N = 117 patients (PAD analysis) Mean age: 73 years Gender: 63% male **Subgroup analysis** Diabetes (72 patients) Mean age: 72 years Gender: 65% male	ABI ≤ 0·90 or > 1·4 TBI ≤ 0·70 Continuous-Wave Doppler (CWD), considered positive if monophasic pattern in either the dorsalis pedis or posterior tibial arteries, demonstrated by low-resistance, slow systolic acceleration and no diastolic flow reversal	DUS (PAD was defined as >50% stenosis)	*Current results only include the subgroup analysis.* **ABI:** Sens: 45·2% Spec: 92·7% PLR: 6·17 NLR: 0·59 **TBI:** Sens: 63·6% Spec: 82·1% PLR: 3·55 NLR: 0·44		**CWD:** Sens: 74·2% Spec: 92·9% PLR: 10·39 NLR: 0·28	Ten percent of patients with diabetes had incompressible ankle pressures.
Tehan et al.^48^ 2017	Australia	Single-centerretrospective case-control study	**Overall** N = 394 participants (suspected PAD) Mean age: 77 years Gender: 61% male **Subgroup analysis** Diabetes (176 patients) Mean age: 75 years Gender: 65% male	Toe pressure < 97 mmHg	DUS (PAD was defined as >50% stenosis)	*Current results only include the subgroup analysis.* **Toe pressure:** Sens: 73·7% Spec: 72·4% PLR: 2·67 NLR: 0·36			TP cutoff value was calculated based on ROC curves. 27% of patients had calcification of peripheral vessels visualised on DUS. However, it was unclear how presence of calcification was assessed.
Tehan et al.^46^ 2018	Australia	Single-centerretrospective case-control study	**Overall** N = 160 patients (278 limbs) with suspected PAD Mean age: 73 years Gender: 69% male **Subgroup analysis** Diabetes (107 patients) Mean age: 71 years Gender: 73% male	ABI ≤ 0·9 Post-exercise ABI: - Post exercise ABI ≤ 0·9 - >20% reduction compared to resting ABI - >30mmHg reduction compared to Resting systolic ankle pressure	DUS (PAD was defined as >50% stenosis)	*Current results only include the subgroup analysis.* **ABI:** Sens: 53·8% Spec: 92·9% PLR: 7·53 NLR: 0·50 **Post-exercise ABI** **(≤ 0·9):** Sens: 69·6% Spec: 80·0% PLR: 3·48 NLR: 0·38		P**ost-exercise** **(≥20%) reduction** **compared to resting ABI:** Sens: 59·6% Spec: 61·1% PLR: 1·53 NLR: 0·66 **Post-exercise** **(≥30mmHg) reduction in systolic ankle pressure:** Sens: 51·1% Spec: 25·0% PLR: 0·68 NLR: 1·96	28% of patients had incompressible ankle pressures. 31% of patients with diabetes had MAC visualised on CDUS. MAC was determined based on DUS, but it is unclear which criteria were used. The PLR/NLR for the *post-exercise* *(>30mmHg) reduction in systolic ankle pressure* were recalculated by the present research group since the PLR/NLR results in the original paper were incorrect.
Tehan et al.^49^ 2018	Australia	Single-center retrospective case-control study	**Overall** N = 396 patients (suspected PAD) Mean age: 77 years Gender: 61% **Subgroup analysis** Diabetes (176 patients) Mean age: 75 years Gender: 65% male **Subgroup analysis** Medial arterial calcification (98 patients) Mean age/gender: not specified	Continuous wave Doppler (CWD): monophasic or absent signal.	DUS (PAD was defined as >50% stenosis)	*Current results only include the subgroup analyses.* **CWD (subgroup DM):** Sens: 82·8% Spec: 88·3% PLR: 7·09 NLR: 0·19 **CWD (subgroup MAC):** Sens: 82·9% Spec: 81·8% PLR: 4·56 NLR: 0·21			Unblinded study MAC was present in 25% of the patients with diabetes. Subgroup analysis of MAC included both patients with and without diabetes. MAC was determined based on DUS, but it is unclear which criteria were used. Biphasic signals were considered as multiphasic (normal).
Ugwu et al.^35^ 2021	Nigeria	Single-center cross-sectional cohort study	N = 163 patients with diabetes (319 legs) with clinical suspicion of lower extremity PAD Mean age: 56 years Gender: 47% male Mean duration of DM was 8·6 years	ABI < 0·9	DUS (PAD was defined as >50%) The severity of stenosis was graded as follows: (1) 50–75% = mild stenosis (2) 76–99% = moderate stenosis (3) complete occlusion = severe stenosis	**ABI (overall):** Sens: 78·46% Spec: 91% PLR: 8·72 NLR: 0·24 **ABI (moderate stenosis):** Sens: 93% Spec: 91% PLR: 10·33 NLR: 0·08		**ABI (mild stenosis):** Sens: 54% Spec: 91% PLR: 6 NLR: 0·51 **ABI (severe stenosis):** Sens: 100% Spec: 91% PLR: 11·11 NLR: 0	Seven patients with ABI >1·3 were excluded. Unclear if study was prospective or retrospective.
*Vriens et al.^36^ 2018	United Kingdom	Single-center prospective cohort study	N = 60 patients with diabetes-related foot ulceration Mean age: 66 years Gender: 75% male Mean duration of DM was 2 years Comorbidity: - 38% CKD	Palpation of pulses ABI <0·9 or >1·3 Ankle pressure: <70 mmHg Toe pressure: <50 mmHg TBI: <= 0·75 TcPO2: < 60mmHg Pole test (the height - in cm - at which the Doppler signal was lost while elevating the leg) Waveform analysis by DUS (monophasic and/or damped waveforms)	DUS (PAD was defined as >50% stenosis)	**Palpation/pulses:** Sens: 55% Spec: 60% PLR: 1·38 NLR: 0·75 **Ankle pressure:** Sens: 47% Spec: 79% PLR: 2·25 NLR: 0·67 **TBI:** Sens: 89% Spec: 45% PLR: 1·62 NLR: 0·24 **Pole test:** Sens: 28% Spec: 97% PLR: 10·29 NLR: 0·74		**ABI:** Sens: 68% Spec: 59% PLR: 1·69 NLR: 0·53 **Toe pressure:** Sens: 45% Spec: 97% PLR: 17·55 NLR: 0·56 **TcPO2:** Sens: 28% Spec: 66% PLR: 0·81 NLR: 1·10 **Waveform:** Sens: 85% Spec: 100% PLR: infinite NLR: 0·15	Waveform analysis was not blinded to the reference test.
Williams et al.^37^ 2005	United Kingdom	Single-center prospective case-control study	**Overall** N = 68 individuals (130 limbs) with diabetes were screened for PAD (without critical ischemia) Mean age/gender: not specified **Subgroup analysis** Diabetes (89 patients) Mean age: 63-69 years Gender: 74% male Mean duration of DM was 11-24 years	Foot pulse: absence of one or both foot pulses. ABI < 0·9 TBI <0·75 Continuous wave Doppler (CWD): loss of triphasic signal.	DUS (PAD was defined as significant velocity change and flow disturbance locally that resulted in loss of reverse flow distally, caused by occlusions or stenosis)	*Current results only include the subgroup analyses.* **Diabetic no neuropathy (n=32 limbs)** **Foot pulse:** Sens: 87% Spec: 53% PLR: 1·85 NLR: 0·25 **TBI:** Sens: 91% Spec: 65% PLR: 2·6 NLR: 7·2 **Diabetic neuropathy (n=57 limbs)** **Foot pulse:** Sens: 81% Spec: 56% PLR: 1·84 NLR: 0·34 **TBI:** Sens: 100% Spec: 61% PLR: 2·56 NLR: 0		**ABI:** Sens: 100% Spec: 88% PLR: 8·33 NLR: 0 **CWD:** Sens: 100% Spec: 92% PLR: 12·5 NLR: 0 **ABI:** Sens: 53% Spec: 95% PLR: 10·6 NLR: 0·49 **CWD:** Sens: 94% Spec: 66% PLR: 2·76 NLR: 0·09	Active foot disease, rest pain, or signs suggestive of lower limb critical ischemia were excluded. The definition of significant velocity change in DUS was not specified.
Zhang et al.^38^ 2010	China	Single-center retrospective case-control study	N = 184 patients with diabetes were screened for PAD Mean age: 63 years Gender: 74% male Mean duration of DM was 11·5 years	ABI < 0·9	DUS (Large plaque>10 mm^2^ with 100% increase in peak systolic velocity)	**ABI:** Sens: 93·75% Spec: 88·16% PLR: 7·92 NLR: 0·07			Patients who had one leg with low ABI and one leg with high ABI were excluded.

Studies of high methodological quality are marked with asterisks (*).

ABI = Ankle-Brachial Index, ABP = Ankle-Brachial Pressure, ACCmax = Maximal Systolic Acceleration, AP = Ankle Pressure, CKD = Chronic Kidney Disease, CTA = Computed Tomography Angiography, CWD = Continuous Wave Doppler, DM = Diabetes Mellitus, DSA = Digital Subtraction Angiography, DUS = Duplex Ultrasonography, MRA = Magnetic Resonance Angiography, PAD = Peripheral Arterial Disease, PI = Pulsatility index, PPG = Photoplethysmography, RPSI = Relative Pulse Slope Index, TBI = Toe-Brachial Index, TcPO2 = Transcutaneous Oxygen Tension, and TP = Toe Pressure.

### Quality assessment of included studies

The results of the quality assessment are illustrated in Table 3. Only three^29^^,^^36^^,^^39^ of the included studies were of high methodological quality (i.e. low risk of bias in all domains assessed). Risk of bias was generally high or unclear with respect to the selection of participants, and the conduct and interpretation of the index tests and reference standards. Applicability concerns were generally low with respect to the selection of patients, the index- and reference tests.

**Table 3:** Methodological assessment of all included studies based on QUADAS-2 tool.

Author & year	Risk of bias				Applicability concerns		
	Patient selection	Index test	Reference standard	Flow & timing	Patient selection	Index test	Reference standard
AbuRahma 2020^40^	Unclear	Low	Unclear	High	Low	Low	Low
Aubert 2013^29^	Low	Low	Low	Low	Low	Low	Low
Buschmann 2018^41^	Low	High	Unclear	High	Low	Low	Low
Clairotte 2009^42^	High	High	High	Low	Low	Low	Low
Faglia Ezio 2010^30^	High	Low	High	Low	Low	Low	Low
Homza 2019^31^	Low	Low	Unclear	Low	Low	Low	Low
Hur 2018^32^	Low	Low	Unclear	Low	Unclear	Low	Low
Janssen 2005^33^	High	Low	Unclear	Low	Low	Low	Unclear
Li 2015^28^	High	High	Unclear	High	Unclear	High	Low
Normahani 2020^39^	Low	Low	Low	Low	Low	Low	Low
Perriss 2005^43^	High	Unclear	Unclear	High	High	Low	Low
Premalatha 2002^34^	High	Low	Unclear	High	Low	Low	Low
Ro 2013^44^	High	Unclear	Unclear	Low	Low	Low	Low
Saunders 2019^27^	High	Unclear	Unclear	Low	Low	Low	Low
Sonter 2017^47^	Low	Unclear	Unclear	Low	Low	Low	Low
Tehan 2016^45^	Low	Low	Unclear	Low	Low	Low	Low
Tehan 2017^48^	High	High	Unclear	Low	Low	Low	Low
Tehan 2018^46^	High	Low	Unclear	Low	Low	Low	Low
Tehan 2018^49^	High	Low	High	Low	Low	Low	Low
Ugwu 2021^35^	Low	Low	Unclear	Low	Low	Low	Low
Vriens 2018^36^	Low	Low	Low	Low	Low	Low	Low
Williams 2005^37^	High	Low	Unclear	Low	High	Low	Low
Zhang 2010^38^	Low	Unclear	Unclear	High	High	Low	Low

H = High = if any of the signaling questions for a domain were answered with ‘no’, potential for bias existed and was graded as high.

L = Low = if all signaling questions for a domain were answered with ‘yes’, the risk of bias was judged as low.

U = Unclear = this category was only used if insufficient data was reported to permit a judgment.

### Ankle-brachial index

Eighteen studies evaluated the ABI to diagnose PAD in patients prone to MAC.^28^^,^^29^^,^^31^, ^32^, ^33^, ^34^, ^35^, ^36^, ^37^, ^38^, ^39^, ^40^, ^41^, ^42^, ^43^, ^44^, ^45^, ^46^ In these studies, 10 different variables were investigated (Table 4 shows an overview). In studies including patients with an ABI >1·3, the PLR ranged between 1·22 and 17, and the NLR ranged between 0 and 0·69 for an ABI with a threshold of <0·90. When an ABI of <0·9 or >1·3 to 1·4 was defined as abnormal, the PLR and NLR ranges changed to 1·69–6·17 and 0·44–0·72, respectively.

**Table 4:** An overview of the different ABI variables to diagnose PAD.

Index test with threshold	ABI >1·3 included/excluded in study population	Number of studies	Number of patients	PLR	NLR	Sensitivity	Specificity
ABI < 0·9^32^^,^^35^	Excluded	2	487	8·72-17	0·24-0·84	17%-78·46%	91%-99%
ABI < 0·9^33^^,^^34^^,^^37^, ^38^, ^39^, ^40^^,^^42^, ^43^, ^44^^,^^46^	Included	10	1801	1·22-17	0-0·69	53%-100%	42%-95%
ABI <0·9 or >1·3-1·4^29^^,^^31^^,^^36^^,^^45^	Included	4	394	1·69-6·17	0·44-0·72	42·3%-68%	59%-92·7%
ABI < 0·88^41^	Included	1	76	3·29	0·53	56%	83%
Oscillometric ABI < 0.9^42^	Included	1	83	7·9	0·74	29%	96%
Oscillometric ABI <0·9 or >1·4^31^	Included	1	62	10·17	0·41	61%	94%
Lower ABI <0·9 or >1·4^31^	Included	1	62	3·63	0·17	87%	76%
ABI >1·45 (Only patient with ABI >1·3 were included)^28^	Included	1	175	4·33	0·41	65%	85%
Post-exercise ABI (≤0·9)^46^	Included	1	107	3·48	0·38	69·6%	80·0%
Post-exercise (>20%) reduction compared to resting ABI^46^	Included	1	107	1·53	0·66	59·6%	61·1%

### Ankle pressure

Three studies mentioned an absolute ankle pressure of <70mmHg as the threshold for diagnosing PAD. In two of these studies, a PLR of 2·25–2·73 and an NLR of 0·67–0·79 were found to detect PAD.^30^^,^^33^^,^^36^ It was not possible to calculate the PLR/NLR as a result of the selection of patients in one study. All included patients had >50% stenosis on DSA, so only the sensitivity of 33% could be given in this study.^30^ A post-exercise reduction of >30mmHg in systolic ankle pressure showed a lower PLR of 0·68 to detect PAD.^46^

### Toe-brachial index; toe pressure

Six studies investigated the TBI as an index test for PAD (with cut-off values of below 0·70 and 0·75).^36^^,^^37^^,^^39^^,^^40^^,^^45^^,^^47^ In these studies, PLRs ranged from 1·62 to 4·26. NLRs fluctuated between 0 and 0·47. In the three studies that evaluated toe pressure, different cut-off values were used.^36^^,^^47^^,^^48^ Vriens et al. used a pressure below 50 mmHg as indicator for PAD, leading to a PLR of 17·55 and an NLR of 0·56.^36^ Sonter et al. studied a pressure below 70 mmHg, with an infinite PLR and an NLR of 0·54.^47^ Tehan et al. used a pressure below 97 mmHg, resulting in a PLR of 2·67 and an NLR of 0·36.^48^

### Palpable pulsations

Four studies explored the palpation of foot pulses as a bedside test.^29^^,^^36^^,^^37^^,^^39^ Since these studies described different criteria for the diagnosis of PAD, these articles will be described separately. Aubert et al. regarded missing or weak foot pulses as an indicator for PAD, leading to a PLR of 2·46 and NLR of 0·43.^29^ Vriens et al. used the absence of foot pulses as PAD criterion, resulting in a PLR of 1·38 and an NLR of 0·75.^36^ Williams et al. and Normahani et al. considered the absence of one or both foot pulses as diagnostic of PAD. This resulted in PLR/NLR of 1.84/0.31 and 2.22/0.71 respectively.^37^^,^^39^

### Waveform analysis

Waveform analysis, measured at the dorsalis pedis- or posterior tibial artery, is described in six articles (two studies investigated two techniques).^36^^,^^37^^,^^39^^,^^44^^,^^45^^,^^49^ Visual waveform analysis was conducted using a Continuous Wave Doppler (CWD) device in five studies, a Duplex ultrasound scanning (DUS) device in two studies, and photoplethysmography in one study. Abnormal waveform was heterogeneously defined. Two studies by Tehan et al. and Normahani et al. described PAD as the presence of a monophasic or dampened waveform using CWD, with a PLR ranging from 3·28 to 10·39 and an NLR of 0·19 to 0·28.^39^^,^^45^^,^^49^ Vriens et al. described an infinite PLR and an NLR of 0·15 for the detection of PAD by DUS waveform analysis, defined as a monophasic or damped waveform. Note that waveform analysis was not blinded to the reference test in this study.^36^ Loss of a triphasic pattern is another parameter for defining PAD and was investigated in two studies using CWD. The PLR varied between 2·76 and 12·5 and the NLR between 0 and 0·09.^37^^,^^44^ The detection of PAD by photoplethysmography waveform assessment showed a PLR of 7·14 and NLR of 0·24.^44^ Normahani et al. investigated the PAD-scan (waveform analysis performed using DUS), this is explained in detail in Table 2. This technique showed a PLR of 4.06 and NLR of 0.07.^39^

### Transcutaneous oxygen pressure

Three studies investigated the reliability of transcutaneous oxygen pressure (TcPO2).^36^^,^^39^ Vriens et al. regarded a pressure below 60 mmHg as PAD, resulting in a PLR of 0·81 and an NLR of 1·10.^36^ Normahani et al. used a pressure below 40 mmHg, which showed a PLR of 1.43 and NLR of 0.88.^39^ Faglia Ezio et al. studied pressures below 30 and 50 mmHg, with a sensitivity of 82% and 100% respectively. Since all patients in this study had PAD (probably due to patient selection), specificity, PLR and NLR could not be calculated.^30^

### Other

Novel arterial Doppler flow parameters, the maximum systolic acceleration (ACCmax) and the relative pulse slope index (RPSI) were explored by Buschmann et al.^41^ The ACCmax, defined as “maximum slope of the velocity curve in the systolic phase” detected PAD with a PLR of 28·5 and NLR of 0·44 when adopting a cut-off value of <4·4m/sec^2^. Janssen et al. described a colour duplex ultrasonography parameter, pulsatility index (PI), as a PAD diagnostic test.^33^ PI is defined as “the ratio of the maximum vertical excursions of the Doppler”, and showed a PLR of 2·29 and NLR of 0·21 for a threshold of <1.2. A pole test, the height in centimeters at which the Doppler signal can no longer be detected while passively elevating the leg, is assessed in one article and showed a PLR 10·29 and NLR 0·74.^36^ Lastly, a study by Saunders et al. described a Vascular Early Warning System device (VEWS).^27^ The VEWS device functions by measuring changes in blood volume in the microvasculature of the foot, as detected by infrared optical sensors. This method showed a PLR of 3·65 and NLR 0·34 when a cut-off of ≤0·94 was selected to detect PAD.

## Discussion

To the best of our knowledge, this is the first systematic review on bedside tests to diagnose PAD in patients prone to MAC. While MAC can hamper the performance of bedside tests to diagnose PAD, only 23 studies investigated the accuracy of bedside tests in patients prone to MAC. Most studies were performed in Western countries, and included predominantly older males with DM. The included studies often contained small study populations and had flaws in methodological quality, raising serious concerns about their reliability. Overall, the performances of the different bedside tests were generally disappointing and highly variable between studies.

Worldwide, the ABI is the most frequently used bedside test to diagnose PAD.^50^ In 18 studies that evaluated the ABI, 10 different ABI variables were investigated (Table 4) in which the ABI threshold or study population (ABI >1·3 included or excluded) differed. In most studies an ABI <0·90 was defined as abnormal, followed by four studies that considered an ABI of <0·90 and >1·3–1·4 as PAD. Two studies investigated an ABI threshold of 0·9 and excluded patients with an ABI >1·3,^32^^,^^35^ which is in line with current guidelines^2^^,^^50^ in which patients with an ABI >1·3–1·4 should undergo alternative tests. In these two studies,^32^^,^^35^ the ABI could accurately rule in PAD with a PLR of 8·72–17, but it failed to rule out PAD (NLR 0·24–0·84). The same pattern was seen in all 18 studies, where 16 studies showed an insufficient NLR >0·2 (small effect on ability to rule out PAD). Generally, including patients with ABI >1·3 resulted in a lower performance to diagnose PAD (PLR 1·22–17). Of note, only one study investigated the use of the lowest ankle pressure to calculate the ABI, which lead to an improved performance of the test (compared to the highest ankle pressure).^31^

Since digital arteries are less affected by MAC, the measurement of toe pressure may be more reliable in patients with DM or CKD. Six studies investigated the use of TBI to diagnose PAD, but none of these studies found a moderate or large effect on the ability to diagnose PAD (PLR > 5).^36^^,^^37^^,^^40^^,^^45^^,^^47^ A mixed performance was seen in the ability to rule out PAD, with NLRs of 0–0·47. However, only one small study (N=57 limbs)^37^ had a large effect on the probability to exclude disease and resulted in this outlier (NLR 0). The other five studies did not have an accurate diagnostic effect to rule out PAD (NLR <0·2).^36^^,^^40^^,^^45^^,^^47^ In the three studies evaluating absolute toe pressure, it was remarkable to note that each study used a different threshold.^36^^,^^47^^,^^48^ A pressure of <50 mmHg appeared to be very accurate in diagnosing PAD (PLR 17·55), but provided poor performance to rule out disease (NLR 0·56).^36^ Raising the cut-off values to 70 and 97 mmHg resulted in a better, however still insufficient, ability to exclude PAD (NLR 0·54 and 0·36).^47^^,^^48^

Palpation of arterial pulsations during physical examination forms another cornerstone of clinical practice. While palpation of arterial pulsations may appear to be an attractive bedside test due to the inexpensive and readily applicable nature, the data supporting this method show limited diagnostic utility.^29^^,^^36^^,^^37^^,^^39^ In these studies, different definitions were regarded as abnormal: I) missing or weak,^29^ II) absence of one or both foot pulses,^37^^,^^39^ and III) absent of pedal pulses.^36^ Either way, deviations in palpation of arterial pulsations showed a poor performance to diagnose PAD in patients prone to MAC (PLR 1·38–2·46).^29^^,^^36^^,^^37^ Moreover, one study made the distinction between the presence (dorsalis pedis artery or posterior tibial artery) and absence of pedal pulses. This study showed that the presence of a palpable pedal pulse was insufficient to exclude PAD (NLR 0·75).^36^

Various other index bedside tests were investigated in the studies included in this review. Visual waveform analysis performed by continuous waveform Doppler (CWD) device showed the best test performance to rule out PAD with a relatively small variation in NLR (0–0·28).^37^^,^^39^^,^^44^^,^^45^^,^^49^ It is important to note that in three of the five studies PAD could not be definitively excluded (NLR >0·2), while three studies demonstrated a moderate to proficient ability to diagnose PAD (PLR >5).^37^^,^^45^^,^^49^ However, the definition of an abnormal test was not consistent between these studies. In three studies, the presence of a monophasic or dampened waveform indicated PAD,^39^^,^^45^^,^^49^ while a loss of a triphasic pattern was described as abnormal in the other two studies.^37^^,^^44^ When a loss of a triphasic pattern was used with CWD, PAD could be accurately ruled out (NLR 0–0·09).^37^^,^^44^ Although very reliable, this cut-off would be hard to implement in daily clinical practice, since the majority of patients prone to MAC have dampened, monophasic, or biphasic waveforms. Therefore, the addition of a loss of triphasic pattern with CWD as criterium for PAD will be of diminished value in clinical practice. Notably, only one of the studies included in this review mentioned the use of audible waveform analysis, with limited performance (PLR 3.04 and NLR 0.35).^39^ The PAD-scan waveform assessment, as described by Normahani et al. seems promising and can accurately rule out PAD (NLR 0.07), however this bedside test is only investigated in one study and could be complex to intepretate.^39^ Furthermore, the evidence supporting the ankle pressure^30^^,^^33^^,^^36^ and TcPO2^30^^,^^36^^,^^39^ as a bedside test in patients with suspected MAC was sparse and poor results were found.

For clinicians, diagnosing PAD in patients with DM or CKD presents a major clinical challenge. Due to comorbidities such as neuropathy, patients frequently have atypical or no symptoms such as ischemic rest pain.^8^ Also, clinical examination provides insufficient reliable information to determine which patients have PAD or need further investigations. Additionally, this review shows that current index tests lack the ability to reliably diagnose or rule out PAD. All these considerations stress the importance of the need for a better bedside test, chiefly since early revascularization in patients with critical limb ischemia is essential to decrease future complications, and minimize morbidity in this patient group.^17^^,^^18^ Moreover, early identification of diabetic patients with PAD is essential to promptly start cardiovascular risk management (CVRM) and thus reduce the risk of events.^15^ It is therefore crucial to have a test that can reliably rule out PAD (i.e. have a low NLR). In this way, the diagnosis is less likely to be missed and more patients will be referred for additional imaging, CVRM, and timely revascularization if necessary. Although this would be the most optimal scenario, it is contrary to currently used tests, in which a high PLR and suboptimal NLR is generally seen.

This systematic review has several limitations. First, the overall methodological quality of the included studies was low. Risk of bias or a concern regarding applicability was present in 20 of the 23 included studies. The QUADAS 2-tool showed a notably high risk of bias regarding patient selection. Additionally, sample sizes were small; in 10 of the included studies less than 100 patients were included. Secondly, the heterogeneity in results was high, with wide ranging PLR and NLR values. Thirdly, data presentation was not uniform across studies exploring a specific technique, and many studies showed a wide variation in index test thresholds. Finally, performing a meta-analysis of the data presented in this review was not possible due to both clinical and methodological heterogeneity. Clinical variation was present due to heterogeneous patient groups (DM versus CKD, infection, age), bedside tests (with corresponding cut-off values and way of measurement), reference test (method and percentage of stenosis defined as PAD) and different exclusion criteria across the studies. Methodological heterogeneity was also present, and included study design (prospective vs. retrospective) and risk of bias (blinding of study). We thus advise tentative interpretation of the results presented in this review, and emphasize the need for standardized research using the QUADAS 2-tool^26^ to establish clinical applicability. Also, future (prospective) studies should focus on ruling out PAD, with emphasis on a homogeneous patient group in which all patients receive the same reference test.

Overall, it remains challenging to rule in or rule out PAD in patients prone to MAC. Based on the results of this systematic review, we counsel against the use of a single bedside test. The ABI (<0·9 and exclusion of >1·3) seems useful to diagnose PAD, and CWD (loss of triphasic pattern) was accurate to rule out PAD. However, the included studies must be interpreted with caution due to serious concerns pertaining to the reliability of these studies and thereby the clinical applicability of the bedside tests explored. Not only more methodologically well-designed studies should be performed, but alternative bedside tests must also be investigated to improve the diagnostic accuracy in patients with MAC.

## Contributors

Literature search: JB, SW, LG, JH, AS

Figure and tables: JB, SW, LG

Study design: JB, SW, LG, JH, AS

Data collection: JB, SW

Data analysis and interpretation: JB, SW, LG, JH, AS

Writing the manuscript: JB, SW, LG

Critical review of manuscript: JH, AS, LG

All authors take full responsibility for the content of the publication.

## Data sharing statement

There is no primary data to share. The utilized search strategy can be found in the supplement and, if wanted, be used to reproduce the extracted articles in the corresponding databases.

## Declaration of interests

We declare no competing interests.

## Appendix Supplementary materials

Image, application 1
