# Supplementary material for: Reliability of bedside tests for diagnosing peripheral arterial disease in patients prone to medial arterial calcification: A systematic review
Source: eClinicalMedicine. 2022 Jul 1;50:101532. doi: 10.1016/j.eclinm.2022.101532 (PMC9256539; doi:10.1016/j.eclinm.2022.101532)
Supplement: Supplementary file 1 [file mmc1.docx]

Supplement SI: Search strategy

In cooperation with a trained librarian (JS), a detailed search strategy was composed. The following databases were searched: PubMed, Embase (OVID-version), Web of Science, Cochrane Library, and Emcare. The query consisted of the combination of the following concepts:

• Peripheral arterial diseases, including DM and CKD

• Bedside non-invasive diagnostic tests (e.g., ABI, TP, TBI)

• Imaging techniques (reference standard)

These concepts were combined using six search strands in order to maximize relevancy and minimize noise. For the different concepts, all relevant keyword variations were used, not only keyword variations in the controlled vocabularies of the various databases, but the free text word variations of these concepts as well. The search strategy was optimized for all consulted databases, taking into account the differences of the various controlled vocabularies as well as the differences of database-specific technical variations (e.g., the use of quotation marks). The search was limited to English language articles. The final search was performed on February 10th, 2021. The bibliographic databases yielded 1016 regular references and 164 meeting abstract references. Full details of the search strategy can be found in the table.

| Database | Search Strategy | Number of references | Number of unique references |
| --- | --- | --- | --- |
| PubMed | ((("Peripheral Arterial Disease"[majr] OR "Peripheral Arterial Disease"[ti] OR "Peripheral Arterial Diseases"[ti] OR "Peripheral Artery Disease"[ti] OR "Peripheral Artery Diseases"[ti] OR "PAD"[ti] OR "peripheral arterial occlusive disease"[ti] OR "peripheral arterial occlusive diseases"[ti] OR "peripheral artery occlusive disease"[ti] OR "peripheral artery occlusive diseases"[ti] OR "Peripheral Vascular Diseases"[majr:noexp] OR "Peripheral Angiopathies"[ti] OR "Peripheral Angiopathy"[ti] OR "Peripheral Arteriopathies"[ti] OR "Peripheral Arteriopathy"[ti] OR "Peripheral Vasculopathies"[ti] OR "Peripheral Vasculopathy"[ti] OR "Monckeberg Medial Calcific Sclerosis"[majr] OR "Monckeberg's Medial Calcific Sclerosis"[ti] OR "Monckeberg's Sclerosis"[ti] OR "Monckeberg Sclerosis"[ti] OR "Monckebergs Sclerosis"[ti] OR "Mönckeberg's Medial Calcific Sclerosis"[ti] OR "Mönckeberg's Sclerosis"[ti] OR "Mönckeberg Sclerosis"[ti] OR "Mönckebergs Sclerosis"[ti] OR "Monckeberg"[ti] OR "Monckeberg*"[ti] OR "Moenckeberg"[ti] OR "Moenckeberg*"[ti] OR "Medial Calcific Sclerosis"[ti] OR "Medial Calcific Scleroses"[ti] OR "Mönckeberg Medial Calcific Sclerosis"[ti] OR "incompressible arter*"[ti] OR "incompressible vessel*"[ti] OR "medial calcified arter*"[ti] OR (("Arterial Occlusive Diseases"[majr] OR "Peripheral Vascular Diseases"[majr]) AND ("peripheral"[ti] OR "peripheral*"[ti] OR "limbs"[ti] OR "limb"[ti] OR "leg"[ti] OR "legs"[ti] OR "extremity"[ti] OR "extremities"[ti] OR "arm"[ti] OR "arms"[ti])) OR "Intermittent Claudication"[majr] OR "Intermittent Claudication"[ti] OR "Leriche Syndrome"[majr] OR "Leriche Syndrome"[ti] OR "Leriche's Syndrome"[ti] OR "Leriches Syndrome"[ti] OR "Arteriosclerosis Obliterans"[majr] OR "Arteriosclerosis Obliterans"[ti] OR (("Atherosclerosis"[majr] OR "Arteriosclerosis"[majr] OR "Atherosclerosis"[ti] OR "Arteriosclerosis"[ti]) AND ("peripheral"[ti] OR "peripheral*"[ti] OR "limbs"[ti] OR "limb"[ti] OR "leg"[ti] OR "legs"[ti] OR "extremity"[ti] OR "extremities"[ti] OR "arm"[ti] OR "arms"[ti]))) AND ("Ankle Brachial Index"[majr] OR "Ankle Brachial Indices"[ti] OR "Ankle-Brachial Index"[ti] OR "Ankle-Brachial Indices"[ti] OR "anklebrachial index"[ti] OR "anklebrachialindex"[ti] OR "ankle brachial"[ti] OR "anklebrachial"[ti] OR "ABI"[ti] OR "Toe Brachial Index"[ti] OR "Toe Brachial Indices"[ti] OR "Toe-Brachial Index"[ti] OR "Toe-Brachial Indices"[ti] OR "Toe Pressure"[ti] OR "toe brachial"[ti] OR "toebrachial"[ti] OR "TBI"[ti] OR "TP"[ti] OR "Oximetry"[majr] OR "Oximetry"[ti] OR "Oximetries"[ti] OR "Oximetr*"[ti] OR "Pulse Oximetries"[ti] OR "Pulse Oximetry"[ti] OR "transcutaneous oxygen tension"[ti] OR "transcutaneous oxygen"[ti] OR "Pulse"[majr] OR "Pulse*"[ti] OR "pulsation"[ti] OR "pulsations"[ti] OR "Blood Gas Monitoring, Transcutaneous"[majr] OR "Transcutaneous Blood Gas Monitoring"[ti] OR "Transcutaneous Capnometry"[ti] OR "PtcO2"[ti] OR "TcPCO2"[ti] OR "Doppler waveform"[ti] OR "Doppler waveforms"[ti] OR "Doppler wave form"[ti] OR "Doppler wave forms"[ti] OR "Ultrasonography, Doppler, Pulsed"[majr:noexp] OR "Pulsed Doppler"[ti] OR "Doppler Pulsed"[ti] OR "non-invasive"[ti] OR "non-invasiv*"[ti] OR "point-of-care test"[ti] OR "point-of-care tests"[ti]) AND ("Angiography"[majr] OR "Angiography"[ti] OR "Angiographies"[ti] OR "Angiogram"[ti] OR "Angiograms"[ti] OR "Angiogra*"[ti] OR "Arteriography"[ti] OR "Arteriographies"[ti] OR "Arteriogra*"[ti] OR "Angiography, Digital Subtraction"[majr] OR "Digital Subtraction Angiography"[ti] OR "DSA"[ti] OR "Magnetic Resonance Angiography"[majr] OR "MRI Angiography"[ti] OR "MRI Angiographies"[ti] OR "Magnetic Resonance Angiography"[ti] OR "Magnetic Resonance Angiographies"[ti] OR "MRA"[ti] OR "Perfusion Magnetic Resonance Imaging"[ti] OR "Perfusion Weighted MRI"[ti] OR "Computed Tomography Angiography"[majr] OR "Computed Tomography Angiography"[ti] OR "Computed Tomography Angiographies"[ti] OR "CT Angiography"[ti] OR "CT Angiographies"[ti] OR "CTA"[ti] OR "Ultrasonography, Doppler, Duplex"[majr:noexp] OR "Duplex Doppler"[ti] OR "Doppler Duplex"[ti] OR "Ultrasonography, Doppler, Color"[majr:noexp] OR "Doppler Color"[ti] OR "Color Doppler"[ti] OR "Doppler Colour"[ti] OR "Colour Doppler"[ti] OR "color duplex"[ti] OR "colour duplex"[ti] OR "duplex color"[ti] OR "duplex colour"[ti] OR "CDUS"[ti] OR "Plethysmography"[majr] OR "Plethysmography"[ti] OR "Plethysmogra*"[ti] OR "color spectral waveforms"[ti] OR "color spectral waveform"[ti] OR "color spectral wave forms"[ti] OR "color spectral wave form"[ti] OR "colour spectral waveforms"[ti] OR "colour spectral waveform"[ti] OR "colour spectral wave forms"[ti] OR "colour spectral wave form"[ti]) AND ("Sensitivity and Specificity"[mesh] OR "Sensitivity"[tiab] OR "Specificity"[tiab] OR "Predictive Value"[tiab] OR "ROC Curve"[tiab] OR "Signal-To-Noise"[tiab] OR "limit of detection"[tiab] OR "Cohort Studies"[mesh] OR "Follow-Up Studies"[mesh] OR "Longitudinal Studies"[mesh] OR "Prospective Studies"[mesh] OR "Retrospective Studies"[mesh] OR "Cohort"[tiab] OR "Cohorts"[tiab] OR "Follow-Up"[tiab] OR "Longitudinal"[tiab] OR "Prospective"[tiab] OR "Retrospective"[tiab] OR "Cross-Sectional Studies"[mesh] OR "Cross-Sectional"[tiab] OR "Observational Study"[pt] OR "Observational Studies as Topic"[mesh] OR "Observational Study"[tiab] OR "effectiveness"[tiab] OR "efficacy"[tiab])) OR (("Peripheral Arterial Disease"[majr] OR "Peripheral Arterial Disease"[ti] OR "Peripheral Arterial Diseases"[ti] OR "Peripheral Artery Disease"[ti] OR "Peripheral Artery Diseases"[ti] OR "PAD"[ti] OR "peripheral arterial occlusive disease"[ti] OR "peripheral arterial occlusive diseases"[ti] OR "peripheral artery occlusive disease"[ti] OR "peripheral artery occlusive diseases"[ti] OR "Peripheral Vascular Diseases"[majr:noexp] OR "Peripheral Angiopathies"[ti] OR "Peripheral Angiopathy"[ti] OR "Peripheral Arteriopathies"[ti] OR "Peripheral Arteriopathy"[ti] OR "Peripheral Vasculopathies"[ti] OR "Peripheral Vasculopathy"[ti] OR "Monckeberg Medial Calcific Sclerosis"[majr] OR "Monckeberg's Medial Calcific Sclerosis"[ti] OR "Monckeberg's Sclerosis"[ti] OR "Monckeberg Sclerosis"[ti] OR "Monckebergs Sclerosis"[ti] OR "Mönckeberg's Medial Calcific Sclerosis"[ti] OR "Mönckeberg's Sclerosis"[ti] OR "Mönckeberg Sclerosis"[ti] OR "Mönckebergs Sclerosis"[ti] OR "Monckeberg"[ti] OR "Monckeberg*"[ti] OR "Moenckeberg"[ti] OR "Moenckeberg*"[ti] OR "Medial Calcific Sclerosis"[ti] OR "Medial Calcific Scleroses"[ti] OR "Mönckeberg Medial Calcific Sclerosis"[ti] OR "incompressible arter*"[ti] OR "incompressible vessel*"[ti] OR "medial calcified arter*"[ti] OR (("Arterial Occlusive Diseases"[majr] OR "Peripheral Vascular Diseases"[majr]) AND ("peripheral"[ti] OR "peripheral*"[ti] OR "limbs"[ti] OR "limb"[ti] OR "leg"[ti] OR "legs"[ti] OR "extremity"[ti] OR "extremities"[ti] OR "arm"[ti] OR "arms"[ti])) OR "Intermittent Claudication"[majr] OR "Intermittent Claudication"[ti] OR "Leriche Syndrome"[majr] OR "Leriche Syndrome"[ti] OR "Leriche's Syndrome"[ti] OR "Leriches Syndrome"[ti] OR "Arteriosclerosis Obliterans"[majr] OR "Arteriosclerosis Obliterans"[ti] OR (("Atherosclerosis"[majr] OR "Arteriosclerosis"[majr] OR "Atherosclerosis"[ti] OR "Arteriosclerosis"[ti]) AND ("peripheral"[ti] OR "peripheral*"[ti] OR "limbs"[ti] OR "limb"[ti] OR "leg"[ti] OR "legs"[ti] OR "extremity"[ti] OR "extremities"[ti] OR "arm"[ti] OR "arms"[ti]))) AND ("Ankle Brachial Index"[majr] OR "Ankle Brachial Indices"[ti] OR "Ankle-Brachial Index"[ti] OR "Ankle-Brachial Indices"[ti] OR "anklebrachial index"[ti] OR "anklebrachialindex"[ti] OR "ankle brachial"[ti] OR "anklebrachial"[ti] OR "ABI"[ti] OR "Toe Brachial Index"[ti] OR "Toe Brachial Indices"[ti] OR "Toe-Brachial Index"[ti] OR "Toe-Brachial Indices"[ti] OR "Toe Pressure"[ti] OR "toe brachial"[ti] OR "toebrachial"[ti] OR "TBI"[ti] OR "TP"[ti] OR "Oximetry"[majr] OR "Oximetry"[ti] OR "Oximetries"[ti] OR "Oximetr*"[ti] OR "Pulse Oximetries"[ti] OR "Pulse Oximetry"[ti] OR "transcutaneous oxygen tension"[ti] OR "transcutaneous oxygen"[ti] OR "Pulse"[majr] OR "Pulse*"[ti] OR "pulsation"[ti] OR "pulsations"[ti] OR "Blood Gas Monitoring, Transcutaneous"[majr] OR "Transcutaneous Blood Gas Monitoring"[ti] OR "Transcutaneous Capnometry"[ti] OR "PtcO2"[ti] OR "TcPCO2"[ti] OR "Doppler waveform"[ti] OR "Doppler waveforms"[ti] OR "Doppler wave form"[ti] OR "Doppler wave forms"[ti] OR "Ultrasonography, Doppler, Pulsed"[majr:noexp] OR "Pulsed Doppler"[ti] OR "Doppler Pulsed"[ti] OR "non-invasive"[ti] OR "non-invasiv*"[ti]) AND ("Sensitivity and Specificity"[majr] OR "Sensitivity"[tiab] OR "Specificity"[tiab] OR "Predictive Value"[tiab] OR "ROC Curve"[tiab] OR "Signal-To-Noise"[tiab] OR "limit of detection"[tiab] OR "Cohort Studies"[majr] OR "Follow-Up Studies"[majr] OR "Longitudinal Studies"[majr] OR "Prospective Studies"[majr] OR "Retrospective Studies"[majr] OR "Cohort"[tiab] OR "Cohorts"[tiab] OR "Follow-Up"[tiab] OR "Longitudinal"[tiab] OR "Prospective"[tiab] OR "Retrospective"[tiab] OR "Cross-Sectional Studies"[majr] OR "Cross-Sectional"[tiab] OR "Observational Study"[pt] OR "Observational Studies as Topic"[majr] OR "Observational Study"[tiab] OR "effectiveness"[tiab] OR "efficacy"[tiab])) OR (("Peripheral Arterial Disease"[majr] OR "Peripheral Arterial Disease"[ti] OR "Peripheral Arterial Diseases"[ti] OR "Peripheral Artery Disease"[ti] OR "Peripheral Artery Diseases"[ti] OR "PAD"[ti] OR "peripheral arterial occlusive disease"[ti] OR "peripheral arterial occlusive diseases"[ti] OR "peripheral artery occlusive disease"[ti] OR "peripheral artery occlusive diseases"[ti] OR "Peripheral Vascular Diseases"[majr:noexp] OR "Peripheral Angiopathies"[ti] OR "Peripheral Angiopathy"[ti] OR "Peripheral Arteriopathies"[ti] OR "Peripheral Arteriopathy"[ti] OR "Peripheral Vasculopathies"[ti] OR "Peripheral Vasculopathy"[ti] OR "Monckeberg Medial Calcific Sclerosis"[majr] OR "Monckeberg's Medial Calcific Sclerosis"[ti] OR "Monckeberg's Sclerosis"[ti] OR "Monckeberg Sclerosis"[ti] OR "Monckebergs Sclerosis"[ti] OR "Mönckeberg's Medial Calcific Sclerosis"[ti] OR "Mönckeberg's Sclerosis"[ti] OR "Mönckeberg Sclerosis"[ti] OR "Mönckebergs Sclerosis"[ti] OR "Monckeberg"[ti] OR "Monckeberg*"[ti] OR "Moenckeberg"[ti] OR "Moenckeberg*"[ti] OR "Medial Calcific Sclerosis"[ti] OR "Medial Calcific Scleroses"[ti] OR "Mönckeberg Medial Calcific Sclerosis"[ti] OR "incompressible arter*"[ti] OR "incompressible vessel*"[ti] OR "medial calcified arter*"[ti] OR (("Arterial Occlusive Diseases"[majr] OR "Peripheral Vascular Diseases"[majr]) AND ("peripheral"[ti] OR "peripheral*"[ti] OR "limbs"[ti] OR "limb"[ti] OR "leg"[ti] OR "legs"[ti] OR "extremity"[ti] OR "extremities"[ti] OR "arm"[ti] OR "arms"[ti])) OR "Intermittent Claudication"[majr] OR "Intermittent Claudication"[ti] OR "Leriche Syndrome"[majr] OR "Leriche Syndrome"[ti] OR "Leriche's Syndrome"[ti] OR "Leriches Syndrome"[ti] OR "Arteriosclerosis Obliterans"[majr] OR "Arteriosclerosis Obliterans"[ti] OR (("Atherosclerosis"[majr] OR "Arteriosclerosis"[majr] OR "Atherosclerosis"[ti] OR "Arteriosclerosis"[ti]) AND ("peripheral"[ti] OR "peripheral*"[ti] OR "limbs"[ti] OR "limb"[ti] OR "leg"[ti] OR "legs"[ti] OR "extremity"[ti] OR "extremities"[ti] OR "arm"[ti] OR "arms"[ti]))) AND ("Ankle Brachial Index"[majr] OR "Ankle Brachial Indices"[ti] OR "Ankle-Brachial Index"[ti] OR "Ankle-Brachial Indices"[ti] OR "anklebrachial index"[ti] OR "anklebrachialindex"[ti] OR "ankle brachial"[ti] OR "anklebrachial"[ti] OR "ABI"[ti] OR "Toe Brachial Index"[ti] OR "Toe Brachial Indices"[ti] OR "Toe-Brachial Index"[ti] OR "Toe-Brachial Indices"[ti] OR "Toe Pressure"[ti] OR "toe brachial"[ti] OR "toebrachial"[ti] OR "TBI"[ti] OR "TP"[ti] OR "Oximetry"[majr] OR "Oximetry"[ti] OR "Oximetries"[ti] OR "Oximetr*"[ti] OR "Pulse Oximetries"[ti] OR "Pulse Oximetry"[ti] OR "transcutaneous oxygen tension"[ti] OR "transcutaneous oxygen"[ti] OR "Pulse"[majr] OR "Pulse*"[ti] OR "pulsation"[ti] OR "pulsations"[ti] OR "Blood Gas Monitoring, Transcutaneous"[majr] OR "Transcutaneous Blood Gas Monitoring"[ti] OR "Transcutaneous Capnometry"[ti] OR "PtcO2"[ti] OR "TcPCO2"[ti] OR "Doppler waveform"[ti] OR "Doppler waveforms"[ti] OR "Doppler wave form"[ti] OR "Doppler wave forms"[ti] OR "Ultrasonography, Doppler, Pulsed"[majr:noexp] OR "Pulsed Doppler"[ti] OR "Doppler Pulsed"[ti] OR "non-invasive"[ti] OR "non-invasiv*"[ti] OR "point-of-care test"[ti] OR "point-of-care tests"[ti] OR "point-of-care test"[ti] OR "point-of-care tests"[ti]) AND ("Angiography"[majr] OR "Angiography"[ti] OR "Angiographies"[ti] OR "Angiogram"[ti] OR "Angiograms"[ti] OR "Angiogra*"[ti] OR "Arteriography"[ti] OR "Arteriographies"[ti] OR "Arteriogra*"[ti] OR "Angiography, Digital Subtraction"[majr] OR "Digital Subtraction Angiography"[ti] OR "DSA"[ti] OR "Magnetic Resonance Angiography"[majr] OR "MRI Angiography"[ti] OR "MRI Angiographies"[ti] OR "Magnetic Resonance Angiography"[ti] OR "Magnetic Resonance Angiographies"[ti] OR "MRA"[ti] OR "Perfusion Magnetic Resonance Imaging"[ti] OR "Perfusion Weighted MRI"[ti] OR "Computed Tomography Angiography"[majr] OR "Computed Tomography Angiography"[ti] OR "Computed Tomography Angiographies"[ti] OR "CT Angiography"[ti] OR "CT Angiographies"[ti] OR "CTA"[ti] OR "Ultrasonography, Doppler, Duplex"[majr:noexp] OR "Duplex Doppler"[ti] OR "Doppler Duplex"[ti] OR "Ultrasonography, Doppler, Color"[majr:noexp] OR "Doppler Color"[ti] OR "Color Doppler"[ti] OR "Doppler Colour"[ti] OR "Colour Doppler"[ti] OR "color duplex"[ti] OR "colour duplex"[ti] OR "Laser-Doppler Flowmetry"[majr] OR "duplex color"[ti] OR "duplex colour"[ti] OR "CDUS"[ti] OR "Plethysmography"[majr] OR "Plethysmography"[ti] OR "Plethysmogra*"[ti] OR "color spectral waveforms"[ti] OR "color spectral waveform"[ti] OR "color spectral wave forms"[ti] OR "color spectral wave form"[ti] OR "colour spectral waveforms"[ti] OR "colour spectral waveform"[ti] OR "colour spectral wave forms"[ti] OR "colour spectral wave form"[ti] OR "Peripheral Vascular Diseases/diagnostic imaging"[majr:noexp] OR "Comparative Study"[pt]) AND ("Monckeberg Medial Calcific Sclerosis"[Mesh] OR "Monckeberg's Medial Calcific Sclerosis"[tw] OR "Monckeberg's Sclerosis"[tw] OR "Monckeberg Sclerosis"[tw] OR "Monckebergs Sclerosis"[tw] OR "Mönckeberg's Medial Calcific Sclerosis"[tw] OR "Mönckeberg's Sclerosis"[tw] OR "Mönckeberg Sclerosis"[tw] OR "Mönckebergs Sclerosis"[tw] OR "Monckeberg"[tw] OR "Monckeberg*"[tw] OR "Moenckeberg"[tw] OR "Moenckeberg*"[tw] OR "Medial Calcific Sclerosis"[tw] OR "Medial Calcific Scleroses"[tw] OR "Mönckeberg Medial Calcific Sclerosis"[tw] OR "incompressible arter*"[tw] OR "incompressible vessel*"[tw] OR "medial calcified arter*"[tw] OR "media sclerosis"[tw] OR "mediasclerosis"[tw] OR "in-compressible artery"[tw] OR "incompressible artery"[tw] OR "in-compressible arteries"[tw] OR "incompressible arteries"[tw] OR "in-compressible vessel"[tw] OR "incompressible vessel"[tw] OR "in-compressible vessels"[tw] OR "incompressible vessels"[tw] OR "non-compressible artery"[tw] OR "noncompressible artery"[tw] OR "non-compressible arteries"[tw] OR "noncompressible arteries"[tw] OR "non-compressible vessel"[tw] OR "noncompressible vessel"[tw] OR "non-compressible vessels"[tw] OR "noncompressible vessels"[tw] OR "in compress*"[tw] OR "incompress*"[tw] OR "non compress*"[tw] OR "noncompress*"[tw] OR "calcifications"[tw] OR "calcification"[tw] OR "calcificat*"[tw] OR "Calcification, Physiologic"[Mesh:NoExp])) OR (("Peripheral Arterial Disease"[majr] OR "Peripheral Arterial Disease"[ti] OR "Peripheral Arterial Diseases"[ti] OR "Peripheral Artery Disease"[ti] OR "Peripheral Artery Diseases"[ti] OR "PAD"[ti] OR "peripheral arterial occlusive disease"[ti] OR "peripheral arterial occlusive diseases"[ti] OR "peripheral artery occlusive disease"[ti] OR "peripheral artery occlusive diseases"[ti] OR "Peripheral Vascular Diseases"[majr:noexp] OR "Peripheral Angiopathies"[ti] OR "Peripheral Angiopathy"[ti] OR "Peripheral Arteriopathies"[ti] OR "Peripheral Arteriopathy"[ti] OR "Peripheral Vasculopathies"[ti] OR "Peripheral Vasculopathy"[ti] OR "Monckeberg Medial Calcific Sclerosis"[majr] OR "Monckeberg's Medial Calcific Sclerosis"[ti] OR "Monckeberg's Sclerosis"[ti] OR "Monckeberg Sclerosis"[ti] OR "Monckebergs Sclerosis"[ti] OR "Mönckeberg's Medial Calcific Sclerosis"[ti] OR "Mönckeberg's Sclerosis"[ti] OR "Mönckeberg Sclerosis"[ti] OR "Mönckebergs Sclerosis"[ti] OR "Monckeberg"[ti] OR "Monckeberg*"[ti] OR "Moenckeberg"[ti] OR "Moenckeberg*"[ti] OR "Medial Calcific Sclerosis"[ti] OR "Medial Calcific Scleroses"[ti] OR "Mönckeberg Medial Calcific Sclerosis"[ti] OR "incompressible arter*"[ti] OR "incompressible vessel*"[ti] OR "medial calcified arter*"[ti] OR (("Arterial Occlusive Diseases"[majr] OR "Peripheral Vascular Diseases"[majr]) AND ("peripheral"[ti] OR "peripheral*"[ti] OR "limbs"[ti] OR "limb"[ti] OR "leg"[ti] OR "legs"[ti] OR "extremity"[ti] OR "extremities"[ti] OR "arm"[ti] OR "arms"[ti])) OR "Intermittent Claudication"[majr] OR "Intermittent Claudication"[ti] OR "Leriche Syndrome"[majr] OR "Leriche Syndrome"[ti] OR "Leriche's Syndrome"[ti] OR "Leriches Syndrome"[ti] OR "Arteriosclerosis Obliterans"[majr] OR "Arteriosclerosis Obliterans"[ti] OR (("Atherosclerosis"[majr] OR "Arteriosclerosis"[majr] OR "Atherosclerosis"[ti] OR "Arteriosclerosis"[ti]) AND ("peripheral"[ti] OR "peripheral*"[ti] OR "limbs"[ti] OR "limb"[ti] OR "leg"[ti] OR "legs"[ti] OR "extremity"[ti] OR "extremities"[ti] OR "arm"[ti] OR "arms"[ti]))) AND ("clinical test"[ti] OR "clinical tests"[ti] OR "screening test"[ti] OR "screening tests"[ti] OR "point-of-care test"[ti] OR "point-of-care tests"[ti])) OR (("Ankle Brachial Index"[majr] OR "Ankle Brachial Indices"[ti] OR "Ankle-Brachial Index"[ti] OR "Ankle-Brachial Indices"[ti] OR "anklebrachial index"[ti] OR "anklebrachialindex"[ti] OR "ankle brachial"[ti] OR "anklebrachial"[ti] OR "ABI"[ti] OR "Toe Brachial Index"[ti] OR "Toe Brachial Indices"[ti] OR "Toe-Brachial Index"[ti] OR "Toe-Brachial Indices"[ti] OR "Toe Pressure"[ti] OR "toe brachial"[ti] OR "toebrachial"[ti] OR "TBI"[ti] OR "TP"[ti] OR "Oximetry"[majr] OR "Oximetry"[ti] OR "Oximetries"[ti] OR "Oximetr*"[ti] OR "Pulse Oximetries"[ti] OR "Pulse Oximetry"[ti] OR "transcutaneous oxygen tension"[ti] OR "transcutaneous oxygen"[ti] OR "Pulse"[majr] OR "Pulse*"[ti] OR "pulsation"[ti] OR "pulsations"[ti] OR "Blood Gas Monitoring, Transcutaneous"[majr] OR "Transcutaneous Blood Gas Monitoring"[ti] OR "Transcutaneous Capnometry"[ti] OR "PtcO2"[ti] OR "TcPCO2"[ti] OR "Doppler waveform"[ti] OR "Doppler waveforms"[ti] OR "Doppler wave form"[ti] OR "Doppler wave forms"[ti] OR "Ultrasonography, Doppler, Pulsed"[majr:noexp] OR "Pulsed Doppler"[ti] OR "Doppler Pulsed"[ti] OR "non-invasive"[ti] OR "non-invasiv*"[ti] OR "point-of-care test"[ti] OR "point-of-care tests"[ti]) AND ("Angiography"[majr] OR "Angiography"[ti] OR "Angiographies"[ti] OR "Angiogram"[ti] OR "Angiograms"[ti] OR "Angiogra*"[ti] OR "Arteriography"[ti] OR "Arteriographies"[ti] OR "Arteriogra*"[ti] OR "Angiography, Digital Subtraction"[majr] OR "Digital Subtraction Angiography"[ti] OR "DSA"[ti] OR "Magnetic Resonance Angiography"[majr] OR "MRI Angiography"[ti] OR "MRI Angiographies"[ti] OR "Magnetic Resonance Angiography"[ti] OR "Magnetic Resonance Angiographies"[ti] OR "MRA"[ti] OR "Perfusion Magnetic Resonance Imaging"[ti] OR "Perfusion Weighted MRI"[ti] OR "Computed Tomography Angiography"[majr] OR "Computed Tomography Angiography"[ti] OR "Computed Tomography Angiographies"[ti] OR "CT Angiography"[ti] OR "CT Angiographies"[ti] OR "CTA"[ti] OR "Ultrasonography, Doppler, Duplex"[majr:noexp] OR "Duplex Doppler"[ti] OR "Doppler Duplex"[ti] OR "Ultrasonography, Doppler, Color"[majr:noexp] OR "Doppler Color"[ti] OR "Color Doppler"[ti] OR "Doppler Colour"[ti] OR "Colour Doppler"[ti] OR "color duplex"[ti] OR "colour duplex"[ti] OR "duplex color"[ti] OR "duplex colour"[ti] OR "CDUS"[ti] OR "Plethysmography"[majr] OR "Plethysmography"[ti] OR "Plethysmogra*"[ti] OR "color spectral waveforms"[ti] OR "color spectral waveform"[ti] OR "color spectral wave forms"[ti] OR "color spectral wave form"[ti] OR "colour spectral waveforms"[ti] OR "colour spectral waveform"[ti] OR "colour spectral wave forms"[ti] OR "colour spectral wave form"[ti]) AND ("Diabetes Mellitus"[majr] OR "diabetes"[ti] OR "diabet*"[ti] OR "Renal Insufficiency, Chronic"[majr] OR "Chronic Kidney Disease"[ti] OR "Chronic Kidney Diseases"[ti] OR "Chronic Kidney Failure"[ti] OR "Chronic Kidney Insufficiency"[ti] OR "Chronic Renal Disease"[ti] OR "Chronic Renal Diseases"[ti] OR "Chronic Renal Failure"[ti] OR "Chronic Renal Insufficiency"[ti] OR "End Stage Kidney Disease"[ti] OR "End Stage Renal Disease"[ti] OR "End-Stage Renal Failure"[ti] OR "End-Stage Kidney Failure"[ti] OR "ESRD"[ti])) OR (("Peripheral Arterial Disease"[majr] OR "Peripheral Arterial Disease"[ti] OR "Peripheral Arterial Diseases"[ti] OR "Peripheral Artery Disease"[ti] OR "Peripheral Artery Diseases"[ti] OR "PAD"[ti] OR "peripheral arterial occlusive disease"[ti] OR "peripheral arterial occlusive diseases"[ti] OR "peripheral artery occlusive disease"[ti] OR "peripheral artery occlusive diseases"[ti] OR "Peripheral Vascular Diseases"[majr:noexp] OR "Peripheral Angiopathies"[ti] OR "Peripheral Angiopathy"[ti] OR "Peripheral Arteriopathies"[ti] OR "Peripheral Arteriopathy"[ti] OR "Peripheral Vasculopathies"[ti] OR "Peripheral Vasculopathy"[ti] OR "Monckeberg Medial Calcific Sclerosis"[majr] OR "Monckeberg's Medial Calcific Sclerosis"[ti] OR "Monckeberg's Sclerosis"[ti] OR "Monckeberg Sclerosis"[ti] OR "Monckebergs Sclerosis"[ti] OR "Mönckeberg's Medial Calcific Sclerosis"[ti] OR "Mönckeberg's Sclerosis"[ti] OR "Mönckeberg Sclerosis"[ti] OR "Mönckebergs Sclerosis"[ti] OR "Monckeberg"[ti] OR "Monckeberg*"[ti] OR "Moenckeberg"[ti] OR "Moenckeberg*"[ti] OR "Medial Calcific Sclerosis"[ti] OR "Medial Calcific Scleroses"[ti] OR "Mönckeberg Medial Calcific Sclerosis"[ti] OR "incompressible arter*"[ti] OR "incompressible vessel*"[ti] OR "medial calcified arter*"[ti] OR (("Arterial Occlusive Diseases"[majr] OR "Peripheral Vascular Diseases"[majr]) AND ("peripheral"[ti] OR "peripheral*"[ti] OR "limbs"[ti] OR "limb"[ti] OR "leg"[ti] OR "legs"[ti] OR "extremity"[ti] OR "extremities"[ti] OR "arm"[ti] OR "arms"[ti])) OR "Intermittent Claudication"[majr] OR "Intermittent Claudication"[ti] OR "Leriche Syndrome"[majr] OR "Leriche Syndrome"[ti] OR "Leriche's Syndrome"[ti] OR "Leriches Syndrome"[ti] OR "Arteriosclerosis Obliterans"[majr] OR "Arteriosclerosis Obliterans"[ti] OR (("Atherosclerosis"[majr] OR "Arteriosclerosis"[majr] OR "Atherosclerosis"[ti] OR "Arteriosclerosis"[ti] OR "ischemia"[ti] OR "ischaemia"[ti]) AND ("peripheral"[ti] OR "peripheral*"[ti] OR "limbs"[ti] OR "limb"[ti] OR "leg"[ti] OR "legs"[ti] OR "extremity"[ti] OR "extremities"[ti] OR "arm"[ti] OR "arms"[ti]))) AND ("Diabetes Mellitus"[majr] OR "diabetes"[ti] OR "diabet*"[ti] OR "Renal Insufficiency, Chronic"[majr] OR "Chronic Kidney Disease"[ti] OR "Chronic Kidney Diseases"[ti] OR "Chronic Kidney Failure"[ti] OR "Chronic Kidney Insufficiency"[ti] OR "Chronic Renal Disease"[ti] OR "Chronic Renal Diseases"[ti] OR "Chronic Renal Failure"[ti] OR "Chronic Renal Insufficiency"[ti] OR "End Stage Kidney Disease"[ti] OR "End Stage Renal Disease"[ti] OR "End-Stage Renal Failure"[ti] OR "End-Stage Kidney Failure"[ti] OR "ESRD"[ti]) AND ("Ankle Brachial Index"[majr] OR "Ankle Brachial Indices"[tiab] OR "Ankle-Brachial Index"[tiab] OR "Ankle-Brachial Indices"[tiab] OR "anklebrachial index"[tiab] OR "anklebrachialindex"[tiab] OR "ankle brachial"[tiab] OR "anklebrachial"[tiab] OR "ABI"[tiab] OR "Toe Brachial Index"[tiab] OR "Toe Brachial Indices"[tiab] OR "Toe-Brachial Index"[tiab] OR "Toe-Brachial Indices"[tiab] OR "Toe Pressure"[tiab] OR "toe brachial"[tiab] OR "toebrachial"[tiab] OR "TBI"[tiab] OR "TP"[tiab] OR "Oximetry"[majr] OR "Oximetry"[tiab] OR "Oximetries"[tiab] OR "Oximetr*"[tiab] OR "Pulse Oximetries"[tiab] OR "Pulse Oximetry"[tiab] OR "transcutaneous oxygen tension"[tiab] OR "transcutaneous oxygen"[tiab] OR "Pulse"[majr] OR "Pulse*"[tiab] OR "pulsation"[tiab] OR "pulsations"[tiab] OR "Blood Gas Monitoring, Transcutaneous"[majr] OR "Transcutaneous Blood Gas Monitoring"[tiab] OR "Transcutaneous Capnometry"[tiab] OR "PtcO2"[tiab] OR "TcPCO2"[tiab] OR "Doppler waveform"[tiab] OR "Doppler waveforms"[tiab] OR "Doppler wave form"[tiab] OR "Doppler wave forms"[tiab] OR "Ultrasonography, Doppler, Pulsed"[majr:noexp] OR "Pulsed Doppler"[tiab] OR "Doppler Pulsed"[tiab] OR "non-invasive"[tiab] OR "non-invasiv*"[tiab] OR "point-of-care test"[tiab] OR "point-of-care tests"[tiab]) AND ("Angiography"[mesh] OR "Angiography"[tiab] OR "Angiographies"[tiab] OR "Angiogram"[tiab] OR "Angiograms"[tiab] OR "Angiogra*"[tiab] OR "Arteriography"[tiab] OR "Arteriographies"[tiab] OR "Arteriogra*"[tiab] OR "Angiography, Digital Subtraction"[mesh] OR "Digital Subtraction Angiography"[tiab] OR "DSA"[tiab] OR "Magnetic Resonance Angiography"[mesh] OR "MRI Angiography"[tiab] OR "MRI Angiographies"[tiab] OR "Magnetic Resonance Angiography"[tiab] OR "Magnetic Resonance Angiographies"[tiab] OR "MRA"[tiab] OR "Perfusion Magnetic Resonance Imaging"[tiab] OR "Perfusion Weighted MRI"[tiab] OR "Computed Tomography Angiography"[mesh] OR "Computed Tomography Angiography"[tiab] OR "Computed Tomography Angiographies"[tiab] OR "CT Angiography"[tiab] OR "CT Angiographies"[tiab] OR "CTA"[tiab] OR "Ultrasonography, Doppler, Duplex"[mesh:noexp] OR "Duplex Doppler"[tiab] OR "Doppler Duplex"[tiab] OR "Ultrasonography, Doppler, Color"[mesh:noexp] OR "Doppler Color"[tiab] OR "Color Doppler"[tiab] OR "Doppler Colour"[tiab] OR "Colour Doppler"[tiab] OR "color duplex"[tiab] OR "colour duplex"[tiab] OR "duplex color"[tiab] OR "duplex colour"[tiab] OR "CDUS"[tiab] OR "Plethysmography"[mesh] OR "Plethysmography"[tiab] OR "Plethysmogra*"[tiab] OR "color spectral waveforms"[tiab] OR "color spectral waveform"[tiab] OR "color spectral wave forms"[tiab] OR "color spectral wave form"[tiab] OR "colour spectral waveforms"[tiab] OR "colour spectral waveform"[tiab] OR "colour spectral wave forms"[tiab] OR "colour spectral wave form"[tiab] OR "Comparative Study"[pt] OR "Cross-Sectional Studies"[mesh] OR "Diagnosis, Computer-Assisted"[mesh] OR "Feasibility Studies"[mesh]))) AND english[la] | 711 | 711 |
|  |  |  |  |
| Embase (OVID-version) | (((*"Peripheral Occlusive Arterial Disease"/ OR "Peripheral Arterial Disease".ti OR "Peripheral Arterial Diseases".ti OR "Peripheral Artery Disease".ti OR "Peripheral Artery Diseases".ti OR "PAD".ti OR "peripheral arterial occlusive disease".ti OR "peripheral arterial occlusive diseases".ti OR "peripheral artery occlusive disease".ti OR "peripheral artery occlusive diseases".ti OR *"Peripheral Vascular Disease"/ OR "Peripheral Angiopathies".ti OR "Peripheral Angiopathy".ti OR "Peripheral Arteriopathies".ti OR "Peripheral Arteriopathy".ti OR "Peripheral Vasculopathies".ti OR "Peripheral Vasculopathy".ti OR *"Monckeberg Medial Calcific Sclerosis"/ OR "Monckeberg's Medial Calcific Sclerosis".ti OR "Monckeberg's Sclerosis".ti OR "Monckeberg Sclerosis".ti OR "Monckebergs Sclerosis".ti OR "Mönckeberg's Medial Calcific Sclerosis".ti OR "Mönckeberg's Sclerosis".ti OR "Mönckeberg Sclerosis".ti OR "Mönckebergs Sclerosis".ti OR "Monckeberg".ti OR "Monckeberg*".ti OR "Moenckeberg".ti OR "Moenckeberg*".ti OR "Medial Calcific Sclerosis".ti OR "Medial Calcific Scleroses".ti OR "Mönckeberg Medial Calcific Sclerosis".ti OR "incompressible arter*".ti OR "incompressible vessel*".ti OR "medial calcified arter*".ti OR ((exp *"Peripheral Occlusive Arterial Disease"/ OR exp *"Peripheral Vascular Disease"/) AND ("peripheral".ti OR "peripheral*".ti OR "limbs".ti OR "limb".ti OR "leg".ti OR "legs".ti OR "extremity".ti OR "extremities".ti OR "arm".ti OR "arms".ti)) OR "Intermittent Claudication"/ OR "Intermittent Claudication".ti OR "Leriche Syndrome"/ OR "Leriche Syndrome".ti OR "Leriche's Syndrome".ti OR "Leriches Syndrome".ti OR "Arteriosclerosis Obliterans".ti OR ((exp *"Atherosclerosis"/ OR exp *"Arteriosclerosis"/ OR "Atherosclerosis".ti OR "Arteriosclerosis".ti) AND ("peripheral".ti OR "peripheral*".ti OR "limbs".ti OR "limb".ti OR "leg".ti OR "legs".ti OR "extremity".ti OR "extremities".ti OR "arm".ti OR "arms".ti))) AND (*"Ankle Brachial Index"/ OR "Ankle Brachial Indices".ti OR "Ankle-Brachial Index".ti OR "Ankle-Brachial Indices".ti OR "anklebrachial index".ti OR "anklebrachialindex".ti OR "ankle brachial".ti OR "anklebrachial".ti OR "ABI".ti OR "Toe Brachial Index".ti OR "Toe Brachial Indices".ti OR "Toe-Brachial Index".ti OR "Toe-Brachial Indices".ti OR "Toe Pressure".ti OR "toe brachial".ti OR "toebrachial".ti OR "TBI".ti OR "TP".ti OR exp *"Oximetry"/ OR "Oximetry".ti OR "Oximetries".ti OR "Oximetr*".ti OR "Pulse Oximetries".ti OR "Pulse Oximetry".ti OR "transcutaneous oxygen tension".ti OR "transcutaneous oxygen".ti OR *"Pulse"/ OR "Pulse*".ti OR "pulsation".ti OR "pulsations".ti OR *"Transcutaneous Oxygen Monitoring"/ OR "Transcutaneous Blood Gas Monitoring".ti OR "Transcutaneous Capnometry".ti OR "PtcO2".ti OR "TcPCO2".ti OR "Doppler waveform".ti OR "Doppler waveforms".ti OR "Doppler wave form".ti OR "Doppler wave forms".ti OR *"pulsed Doppler echocardiography"/ OR "Pulsed Doppler".ti OR "Doppler Pulsed".ti OR "non-invasive".ti OR "non-invasiv*".ti OR "point-of-care test".ti OR "point-of-care tests".ti) AND (exp *"Angiography"/ OR "Angiography".ti OR "Angiographies".ti OR "Angiogram".ti OR "Angiograms".ti OR "Angiogra*".ti OR "Arteriography".ti OR "Arteriographies".ti OR "Arteriogra*".ti OR "Digital Subtraction Angiography".ti OR "DSA".ti OR exp *"Magnetic Resonance Angiography"/ OR "MRI Angiography".ti OR "MRI Angiographies".ti OR "Magnetic Resonance Angiography".ti OR "Magnetic Resonance Angiographies".ti OR "MRA".ti OR *"perfusion weighted imaging"/ OR "Perfusion Magnetic Resonance Imaging".ti OR "Perfusion Weighted MRI".ti OR *"Computed Tomographic Angiography"/ OR "Computed Tomography Angiography".ti OR "Computed Tomography Angiographies".ti OR "CT Angiography".ti OR "CT Angiographies".ti OR "CTA".ti OR *"duplex Doppler ultrasonography"/ OR "Duplex Doppler".ti OR "Doppler Duplex".ti OR *"color Doppler flowmetry"/ OR "Doppler Color".ti OR "Color Doppler".ti OR "Doppler Colour".ti OR "Colour Doppler".ti OR "color duplex".ti OR "colour duplex".ti OR "duplex color".ti OR "duplex colour".ti OR "CDUS".ti OR exp *"Plethysmography"/ OR "Plethysmography".ti OR "Plethysmogra*".ti OR "color spectral waveforms".ti OR "color spectral waveform".ti OR "color spectral wave forms".ti OR "color spectral wave form".ti OR "colour spectral waveforms".ti OR "colour spectral waveform".ti OR "colour spectral wave forms".ti OR "colour spectral wave form".ti) AND ("Sensitivity and Specificity"/ OR "Sensitivity".ti,ab OR "Specificity".ti,ab OR "Predictive Value".ti,ab OR "ROC Curve".ti,ab OR "Signal-To-Noise".ti,ab OR "limit of detection".ti,ab OR "Cohort Analysis"/ OR exp "Follow Up"/ OR exp "Longitudinal Study"/ OR exp "Prospective Study"/ OR exp "Retrospective Study"/ OR "Cohort".ti,ab OR "Cohorts".ti,ab OR "Follow-Up".ti,ab OR "Longitudinal".ti,ab OR "Prospective".ti,ab OR "Retrospective".ti,ab OR exp "Cross-Sectional Study"/ OR "Cross-Sectional".ti,ab OR "Observational Study"/ OR "Observational Study".ti,ab OR "effectiveness".ti,ab OR "efficacy".ti,ab)) OR ((*"Peripheral Occlusive Arterial Disease"/ OR "Peripheral Arterial Disease".ti OR "Peripheral Arterial Diseases".ti OR "Peripheral Artery Disease".ti OR "Peripheral Artery Diseases".ti OR "PAD".ti OR "peripheral arterial occlusive disease".ti OR "peripheral arterial occlusive diseases".ti OR "peripheral artery occlusive disease".ti OR "peripheral artery occlusive diseases".ti OR *"Peripheral Vascular Disease"/ OR "Peripheral Angiopathies".ti OR "Peripheral Angiopathy".ti OR "Peripheral Arteriopathies".ti OR "Peripheral Arteriopathy".ti OR "Peripheral Vasculopathies".ti OR "Peripheral Vasculopathy".ti OR *"Monckeberg Medial Calcific Sclerosis"/ OR "Monckeberg's Medial Calcific Sclerosis".ti OR "Monckeberg's Sclerosis".ti OR "Monckeberg Sclerosis".ti OR "Monckebergs Sclerosis".ti OR "Mönckeberg's Medial Calcific Sclerosis".ti OR "Mönckeberg's Sclerosis".ti OR "Mönckeberg Sclerosis".ti OR "Mönckebergs Sclerosis".ti OR "Monckeberg".ti OR "Monckeberg*".ti OR "Moenckeberg".ti OR "Moenckeberg*".ti OR "Medial Calcific Sclerosis".ti OR "Medial Calcific Scleroses".ti OR "Mönckeberg Medial Calcific Sclerosis".ti OR "incompressible arter*".ti OR "incompressible vessel*".ti OR "medial calcified arter*".ti OR ((exp *"Peripheral Occlusive Arterial Disease"/ OR exp *"Peripheral Vascular Disease"/) AND ("peripheral".ti OR "peripheral*".ti OR "limbs".ti OR "limb".ti OR "leg".ti OR "legs".ti OR "extremity".ti OR "extremities".ti OR "arm".ti OR "arms".ti)) OR "Intermittent Claudication"/ OR "Intermittent Claudication".ti OR "Leriche Syndrome"/ OR "Leriche Syndrome".ti OR "Leriche's Syndrome".ti OR "Leriches Syndrome".ti OR "Arteriosclerosis Obliterans".ti OR ((exp *"Atherosclerosis"/ OR exp *"Arteriosclerosis"/ OR "Atherosclerosis".ti OR "Arteriosclerosis".ti) AND ("peripheral".ti OR "peripheral*".ti OR "limbs".ti OR "limb".ti OR "leg".ti OR "legs".ti OR "extremity".ti OR "extremities".ti OR "arm".ti OR "arms".ti))) AND (*"Ankle Brachial Index"/ OR "Ankle Brachial Indices".ti OR "Ankle-Brachial Index".ti OR "Ankle-Brachial Indices".ti OR "anklebrachial index".ti OR "anklebrachialindex".ti OR "ankle brachial".ti OR "anklebrachial".ti OR "ABI".ti OR "Toe Brachial Index".ti OR "Toe Brachial Indices".ti OR "Toe-Brachial Index".ti OR "Toe-Brachial Indices".ti OR "Toe Pressure".ti OR "toe brachial".ti OR "toebrachial".ti OR "TBI".ti OR "TP".ti OR exp *"Oximetry"/ OR "Oximetry".ti OR "Oximetries".ti OR "Oximetr*".ti OR "Pulse Oximetries".ti OR "Pulse Oximetry".ti OR "transcutaneous oxygen tension".ti OR "transcutaneous oxygen".ti OR *"Pulse"/ OR "Pulse*".ti OR "pulsation".ti OR "pulsations".ti OR *"Transcutaneous Oxygen Monitoring"/ OR "Transcutaneous Blood Gas Monitoring".ti OR "Transcutaneous Capnometry".ti OR "PtcO2".ti OR "TcPCO2".ti OR "Doppler waveform".ti OR "Doppler waveforms".ti OR "Doppler wave form".ti OR "Doppler wave forms".ti OR *"pulsed Doppler echocardiography"/ OR "Pulsed Doppler".ti OR "Doppler Pulsed".ti OR "non-invasive".ti OR "non-invasiv*".ti OR "point-of-care test".ti OR "point-of-care tests".ti) AND (*"Sensitivity and Specificity"/ OR "Sensitivity".ti,ab OR "Specificity".ti,ab OR "Predictive Value".ti,ab OR "ROC Curve".ti,ab OR "Signal-To-Noise".ti,ab OR "limit of detection".ti,ab OR *"Cohort Analysis"/ OR exp "Follow Up"/ OR exp *"Longitudinal Study"/ OR exp *"Prospective Study"/ OR exp *"Retrospective Study"/ OR "Cohort".ti,ab OR "Cohorts".ti,ab OR "Follow-Up".ti,ab OR "Longitudinal".ti,ab OR "Prospective".ti,ab OR "Retrospective".ti,ab OR exp *"Cross-Sectional Study"/ OR "Cross-Sectional".ti,ab OR *"Observational Study"/ OR "Observational Study".ti,ab OR "effectiveness".ti,ab OR "efficacy".ti,ab)) OR ((*"Peripheral Occlusive Arterial Disease"/ OR "Peripheral Arterial Disease".ti OR "Peripheral Arterial Diseases".ti OR "Peripheral Artery Disease".ti OR "Peripheral Artery Diseases".ti OR "PAD".ti OR "peripheral arterial occlusive disease".ti OR "peripheral arterial occlusive diseases".ti OR "peripheral artery occlusive disease".ti OR "peripheral artery occlusive diseases".ti OR *"Peripheral Vascular Disease"/ OR "Peripheral Angiopathies".ti OR "Peripheral Angiopathy".ti OR "Peripheral Arteriopathies".ti OR "Peripheral Arteriopathy".ti OR "Peripheral Vasculopathies".ti OR "Peripheral Vasculopathy".ti OR *"Monckeberg Medial Calcific Sclerosis"/ OR "Monckeberg's Medial Calcific Sclerosis".ti OR "Monckeberg's Sclerosis".ti OR "Monckeberg Sclerosis".ti OR "Monckebergs Sclerosis".ti OR "Mönckeberg's Medial Calcific Sclerosis".ti OR "Mönckeberg's Sclerosis".ti OR "Mönckeberg Sclerosis".ti OR "Mönckebergs Sclerosis".ti OR "Monckeberg".ti OR "Monckeberg*".ti OR "Moenckeberg".ti OR "Moenckeberg*".ti OR "Medial Calcific Sclerosis".ti OR "Medial Calcific Scleroses".ti OR "Mönckeberg Medial Calcific Sclerosis".ti OR "incompressible arter*".ti OR "incompressible vessel*".ti OR "medial calcified arter*".ti OR ((exp *"Peripheral Occlusive Arterial Disease"/ OR exp *"Peripheral Vascular Disease"/) AND ("peripheral".ti OR "peripheral*".ti OR "limbs".ti OR "limb".ti OR "leg".ti OR "legs".ti OR "extremity".ti OR "extremities".ti OR "arm".ti OR "arms".ti)) OR "Intermittent Claudication"/ OR "Intermittent Claudication".ti OR "Leriche Syndrome"/ OR "Leriche Syndrome".ti OR "Leriche's Syndrome".ti OR "Leriches Syndrome".ti OR "Arteriosclerosis Obliterans".ti OR ((exp *"Atherosclerosis"/ OR exp *"Arteriosclerosis"/ OR "Atherosclerosis".ti OR "Arteriosclerosis".ti) AND ("peripheral".ti OR "peripheral*".ti OR "limbs".ti OR "limb".ti OR "leg".ti OR "legs".ti OR "extremity".ti OR "extremities".ti OR "arm".ti OR "arms".ti))) AND (*"Ankle Brachial Index"/ OR "Ankle Brachial Indices".ti OR "Ankle-Brachial Index".ti OR "Ankle-Brachial Indices".ti OR "anklebrachial index".ti OR "anklebrachialindex".ti OR "ankle brachial".ti OR "anklebrachial".ti OR "ABI".ti OR "Toe Brachial Index".ti OR "Toe Brachial Indices".ti OR "Toe-Brachial Index".ti OR "Toe-Brachial Indices".ti OR "Toe Pressure".ti OR "toe brachial".ti OR "toebrachial".ti OR "TBI".ti OR "TP".ti OR exp *"Oximetry"/ OR "Oximetry".ti OR "Oximetries".ti OR "Oximetr*".ti OR "Pulse Oximetries".ti OR "Pulse Oximetry".ti OR "transcutaneous oxygen tension".ti OR "transcutaneous oxygen".ti OR *"Pulse"/ OR "Pulse*".ti OR "pulsation".ti OR "pulsations".ti OR *"Transcutaneous Oxygen Monitoring"/ OR "Transcutaneous Blood Gas Monitoring".ti OR "Transcutaneous Capnometry".ti OR "PtcO2".ti OR "TcPCO2".ti OR "Doppler waveform".ti OR "Doppler waveforms".ti OR "Doppler wave form".ti OR "Doppler wave forms".ti OR *"pulsed Doppler echocardiography"/ OR "Pulsed Doppler".ti OR "Doppler Pulsed".ti OR "non-invasive".ti OR "non-invasiv*".ti OR "point-of-care test".ti OR "point-of-care tests".ti OR "point-of-care test".ti OR "point-of-care tests".ti OR "point-of-care test".ti OR "point-of-care tests".ti) AND (exp *"Angiography"/ OR "Angiography".ti OR "Angiographies".ti OR "Angiogram".ti OR "Angiograms".ti OR "Angiogra*".ti OR "Arteriography".ti OR "Arteriographies".ti OR "Arteriogra*".ti OR "Digital Subtraction Angiography".ti OR "DSA".ti OR exp *"Magnetic Resonance Angiography"/ OR "MRI Angiography".ti OR "MRI Angiographies".ti OR "Magnetic Resonance Angiography".ti OR "Magnetic Resonance Angiographies".ti OR "MRA".ti OR *"perfusion weighted imaging"/ OR "Perfusion Magnetic Resonance Imaging".ti OR "Perfusion Weighted MRI".ti OR *"Computed Tomographic Angiography"/ OR "Computed Tomography Angiography".ti OR "Computed Tomography Angiographies".ti OR "CT Angiography".ti OR "CT Angiographies".ti OR "CTA".ti OR *"duplex Doppler ultrasonography"/ OR "Duplex Doppler".ti OR "Doppler Duplex".ti OR *"color Doppler flowmetry"/ OR "Doppler Color".ti OR "Color Doppler".ti OR "Doppler Colour".ti OR "Colour Doppler".ti OR "color duplex".ti OR "colour duplex".ti OR "duplex color".ti OR "duplex colour".ti OR "CDUS".ti OR exp *"Plethysmography"/ OR "Plethysmography".ti OR "Plethysmogra*".ti OR "color spectral waveforms".ti OR "color spectral waveform".ti OR "color spectral wave forms".ti OR "color spectral wave form".ti OR "colour spectral waveforms".ti OR "colour spectral waveform".ti OR "colour spectral wave forms".ti OR "colour spectral wave form".ti) AND ("Monckeberg Medial Calcific Sclerosis"/ OR "Monckeberg's Medial Calcific Sclerosis".mp OR "Monckeberg's Sclerosis".mp OR "Monckeberg Sclerosis".mp OR "Monckebergs Sclerosis".mp OR "Mönckeberg's Medial Calcific Sclerosis".mp OR "Mönckeberg's Sclerosis".mp OR "Mönckeberg Sclerosis".mp OR "Mönckebergs Sclerosis".mp OR "Monckeberg".mp OR "Monckeberg*".mp OR "Moenckeberg".mp OR "Moenckeberg*".mp OR "Medial Calcific Sclerosis".mp OR "Medial Calcific Scleroses".mp OR "Mönckeberg Medial Calcific Sclerosis".mp OR "incompressible arter*".mp OR "incompressible vessel*".mp OR "medial calcified arter*".mp OR "media sclerosis".mp OR "mediasclerosis".mp OR "in-compressible artery".mp OR "incompressible artery".mp OR "in-compressible arteries".mp OR "incompressible arteries".mp OR "in-compressible vessel".mp OR "incompressible vessel".mp OR "in-compressible vessels".mp OR "incompressible vessels".mp OR "non-compressible artery".mp OR "noncompressible artery".mp OR "non-compressible arteries".mp OR "noncompressible arteries".mp OR "non-compressible vessel".mp OR "noncompressible vessel".mp OR "non-compressible vessels".mp OR "noncompressible vessels".mp OR "in compress*".mp OR "incompress*".mp OR "non compress*".mp OR "noncompress*".mp OR "calcifications".mp OR "calcification".mp OR "calcificat*".mp OR exp *"Calcification"/)) OR ((*"Peripheral Occlusive Arterial Disease"/ OR "Peripheral Arterial Disease".ti OR "Peripheral Arterial Diseases".ti OR "Peripheral Artery Disease".ti OR "Peripheral Artery Diseases".ti OR "PAD".ti OR "peripheral arterial occlusive disease".ti OR "peripheral arterial occlusive diseases".ti OR "peripheral artery occlusive disease".ti OR "peripheral artery occlusive diseases".ti OR *"Peripheral Vascular Disease"/ OR "Peripheral Angiopathies".ti OR "Peripheral Angiopathy".ti OR "Peripheral Arteriopathies".ti OR "Peripheral Arteriopathy".ti OR "Peripheral Vasculopathies".ti OR "Peripheral Vasculopathy".ti OR *"Monckeberg Medial Calcific Sclerosis"/ OR "Monckeberg's Medial Calcific Sclerosis".ti OR "Monckeberg's Sclerosis".ti OR "Monckeberg Sclerosis".ti OR "Monckebergs Sclerosis".ti OR "Mönckeberg's Medial Calcific Sclerosis".ti OR "Mönckeberg's Sclerosis".ti OR "Mönckeberg Sclerosis".ti OR "Mönckebergs Sclerosis".ti OR "Monckeberg".ti OR "Monckeberg*".ti OR "Moenckeberg".ti OR "Moenckeberg*".ti OR "Medial Calcific Sclerosis".ti OR "Medial Calcific Scleroses".ti OR "Mönckeberg Medial Calcific Sclerosis".ti OR "incompressible arter*".ti OR "incompressible vessel*".ti OR "medial calcified arter*".ti OR ((exp *"Peripheral Occlusive Arterial Disease"/ OR exp *"Peripheral Vascular Disease"/) AND ("peripheral".ti OR "peripheral*".ti OR "limbs".ti OR "limb".ti OR "leg".ti OR "legs".ti OR "extremity".ti OR "extremities".ti OR "arm".ti OR "arms".ti)) OR "Intermittent Claudication"/ OR "Intermittent Claudication".ti OR "Leriche Syndrome"/ OR "Leriche Syndrome".ti OR "Leriche's Syndrome".ti OR "Leriches Syndrome".ti OR "Arteriosclerosis Obliterans".ti OR ((exp *"Atherosclerosis"/ OR exp *"Arteriosclerosis"/ OR "Atherosclerosis".ti OR "Arteriosclerosis".ti) AND ("peripheral".ti OR "peripheral*".ti OR "limbs".ti OR "limb".ti OR "leg".ti OR "legs".ti OR "extremity".ti OR "extremities".ti OR "arm".ti OR "arms".ti))) AND ("clinical test".ti OR "clinical tests".ti OR "screening test".ti OR "screening tests".ti OR "point-of-care test".ti OR "point-of-care tests".ti)) OR ((*"Ankle Brachial Index"/ OR "Ankle Brachial Indices".ti OR "Ankle-Brachial Index".ti OR "Ankle-Brachial Indices".ti OR "anklebrachial index".ti OR "anklebrachialindex".ti OR "ankle brachial".ti OR "anklebrachial".ti OR "ABI".ti OR "Toe Brachial Index".ti OR "Toe Brachial Indices".ti OR "Toe-Brachial Index".ti OR "Toe-Brachial Indices".ti OR "Toe Pressure".ti OR "toe brachial".ti OR "toebrachial".ti OR "TBI".ti OR "TP".ti OR exp *"Oximetry"/ OR "Oximetry".ti OR "Oximetries".ti OR "Oximetr*".ti OR "Pulse Oximetries".ti OR "Pulse Oximetry".ti OR "transcutaneous oxygen tension".ti OR "transcutaneous oxygen".ti OR *"Pulse"/ OR "Pulse*".ti OR "pulsation".ti OR "pulsations".ti OR *"Transcutaneous Oxygen Monitoring"/ OR "Transcutaneous Blood Gas Monitoring".ti OR "Transcutaneous Capnometry".ti OR "PtcO2".ti OR "TcPCO2".ti OR "Doppler waveform".ti OR "Doppler waveforms".ti OR "Doppler wave form".ti OR "Doppler wave forms".ti OR *"pulsed Doppler echocardiography"/ OR "Pulsed Doppler".ti OR "Doppler Pulsed".ti OR "non-invasive".ti OR "non-invasiv*".ti OR "point-of-care test".ti OR "point-of-care tests".ti OR "point-of-care test".ti OR "point-of-care tests".ti) AND (exp *"Angiography"/ OR "Angiography".ti OR "Angiographies".ti OR "Angiogram".ti OR "Angiograms".ti OR "Angiogra*".ti OR "Arteriography".ti OR "Arteriographies".ti OR "Arteriogra*".ti OR "Digital Subtraction Angiography".ti OR "DSA".ti OR exp *"Magnetic Resonance Angiography"/ OR "MRI Angiography".ti OR "MRI Angiographies".ti OR "Magnetic Resonance Angiography".ti OR "Magnetic Resonance Angiographies".ti OR "MRA".ti OR *"perfusion weighted imaging"/ OR "Perfusion Magnetic Resonance Imaging".ti OR "Perfusion Weighted MRI".ti OR *"Computed Tomographic Angiography"/ OR "Computed Tomography Angiography".ti OR "Computed Tomography Angiographies".ti OR "CT Angiography".ti OR "CT Angiographies".ti OR "CTA".ti OR *"duplex Doppler ultrasonography"/ OR "Duplex Doppler".ti OR "Doppler Duplex".ti OR *"color Doppler flowmetry"/ OR "Doppler Color".ti OR "Color Doppler".ti OR "Doppler Colour".ti OR "Colour Doppler".ti OR "color duplex".ti OR "colour duplex".ti OR "duplex color".ti OR "duplex colour".ti OR "CDUS".ti OR exp *"Plethysmography"/ OR "Plethysmography".ti OR "Plethysmogra*".ti OR "color spectral waveforms".ti OR "color spectral waveform".ti OR "color spectral wave forms".ti OR "color spectral wave form".ti OR "colour spectral waveforms".ti OR "colour spectral waveform".ti OR "colour spectral wave forms".ti OR "colour spectral wave form".ti) AND (exp *"Diabetes Mellitus"/ OR "diabetes".ti OR "diabet*".ti OR *"Chronic Kidney Failure"/ OR *"end stage renal disease"/ OR "Chronic Kidney Disease".ti OR "Chronic Kidney Diseases".ti OR "Chronic Kidney Failure".ti OR "Chronic Kidney Insufficiency".ti OR "Chronic Renal Disease".ti OR "Chronic Renal Diseases".ti OR "Chronic Renal Failure".ti OR "Chronic Renal Insufficiency".ti OR "End Stage Kidney Disease".ti OR "End Stage Renal Disease".ti OR "End-Stage Renal Failure".ti OR "End-Stage Kidney Failure".ti OR "ESRD".ti)) OR ((*"Peripheral Occlusive Arterial Disease"/ OR "Peripheral Arterial Disease".ti OR "Peripheral Arterial Diseases".ti OR "Peripheral Artery Disease".ti OR "Peripheral Artery Diseases".ti OR "PAD".ti OR "peripheral arterial occlusive disease".ti OR "peripheral arterial occlusive diseases".ti OR "peripheral artery occlusive disease".ti OR "peripheral artery occlusive diseases".ti OR *"Peripheral Vascular Disease"/ OR "Peripheral Angiopathies".ti OR "Peripheral Angiopathy".ti OR "Peripheral Arteriopathies".ti OR "Peripheral Arteriopathy".ti OR "Peripheral Vasculopathies".ti OR "Peripheral Vasculopathy".ti OR *"Monckeberg Medial Calcific Sclerosis"/ OR "Monckeberg's Medial Calcific Sclerosis".ti OR "Monckeberg's Sclerosis".ti OR "Monckeberg Sclerosis".ti OR "Monckebergs Sclerosis".ti OR "Mönckeberg's Medial Calcific Sclerosis".ti OR "Mönckeberg's Sclerosis".ti OR "Mönckeberg Sclerosis".ti OR "Mönckebergs Sclerosis".ti OR "Monckeberg".ti OR "Monckeberg*".ti OR "Moenckeberg".ti OR "Moenckeberg*".ti OR "Medial Calcific Sclerosis".ti OR "Medial Calcific Scleroses".ti OR "Mönckeberg Medial Calcific Sclerosis".ti OR "incompressible arter*".ti OR "incompressible vessel*".ti OR "medial calcified arter*".ti OR ((exp *"Peripheral Occlusive Arterial Disease"/ OR exp *"Peripheral Vascular Disease"/) AND ("peripheral".ti OR "peripheral*".ti OR "limbs".ti OR "limb".ti OR "leg".ti OR "legs".ti OR "extremity".ti OR "extremities".ti OR "arm".ti OR "arms".ti)) OR "Intermittent Claudication"/ OR "Intermittent Claudication".ti OR "Leriche Syndrome"/ OR "Leriche Syndrome".ti OR "Leriche's Syndrome".ti OR "Leriches Syndrome".ti OR "Arteriosclerosis Obliterans".ti OR ((exp *"Atherosclerosis"/ OR exp *"Arteriosclerosis"/ OR "Atherosclerosis".ti OR "Arteriosclerosis".ti) AND ("peripheral".ti OR "peripheral*".ti OR "limbs".ti OR "limb".ti OR "leg".ti OR "legs".ti OR "extremity".ti OR "extremities".ti OR "arm".ti OR "arms".ti))) AND (exp *"Diabetes Mellitus"/ OR "diabetes".ti OR "diabet*".ti OR *"Chronic Kidney Failure"/ OR *"end stage renal disease"/ OR "Chronic Kidney Disease".ti OR "Chronic Kidney Diseases".ti OR "Chronic Kidney Failure".ti OR "Chronic Kidney Insufficiency".ti OR "Chronic Renal Disease".ti OR "Chronic Renal Diseases".ti OR "Chronic Renal Failure".ti OR "Chronic Renal Insufficiency".ti OR "End Stage Kidney Disease".ti OR "End Stage Renal Disease".ti OR "End-Stage Renal Failure".ti OR "End-Stage Kidney Failure".ti OR "ESRD".ti) AND (*"Ankle Brachial Index"/ OR "Ankle Brachial Indices".ti,ab OR "Ankle-Brachial Index".ti,ab OR "Ankle-Brachial Indices".ti,ab OR "anklebrachial index".ti,ab OR "anklebrachialindex".ti,ab OR "ankle brachial".ti,ab OR "anklebrachial".ti,ab OR "ABI".ti,ab OR "Toe Brachial Index".ti,ab OR "Toe Brachial Indices".ti,ab OR "Toe-Brachial Index".ti,ab OR "Toe-Brachial Indices".ti,ab OR "Toe Pressure".ti,ab OR "toe brachial".ti,ab OR "toebrachial".ti,ab OR "TBI".ti,ab OR "TP".ti,ab OR exp *"Oximetry"/ OR "Oximetry".ti,ab OR "Oximetries".ti,ab OR "Oximetr*".ti,ab OR "Pulse Oximetries".ti,ab OR "Pulse Oximetry".ti,ab OR "transcutaneous oxygen tension".ti,ab OR "transcutaneous oxygen".ti,ab OR *"Pulse"/ OR "Pulse*".ti,ab OR "pulsation".ti,ab OR "pulsations".ti,ab OR *"Transcutaneous Oxygen Monitoring"/ OR "Transcutaneous Blood Gas Monitoring".ti,ab OR "Transcutaneous Capnometry".ti,ab OR "PtcO2".ti,ab OR "TcPCO2".ti,ab OR "Doppler waveform".ti,ab OR "Doppler waveforms".ti,ab OR "Doppler wave form".ti,ab OR "Doppler wave forms".ti,ab OR *"pulsed Doppler echocardiography"/ OR "Pulsed Doppler".ti,ab OR "Doppler Pulsed".ti,ab OR "non-invasive".ti,ab OR "non-invasiv*".ti,ab OR "point-of-care test".ti,ab OR "point-of-care tests".ti,ab OR "point-of-care test".ti,ab OR "point-of-care tests".ti,ab) AND (exp *"Angiography"/ OR "Angiography".ti,ab OR "Angiographies".ti,ab OR "Angiogram".ti,ab OR "Angiograms".ti,ab OR "Angiogra*".ti,ab OR "Arteriography".ti,ab OR "Arteriographies".ti,ab OR "Arteriogra*".ti,ab OR "Digital Subtraction Angiography".ti,ab OR "DSA".ti,ab OR exp *"Magnetic Resonance Angiography"/ OR "MRI Angiography".ti,ab OR "MRI Angiographies".ti,ab OR "Magnetic Resonance Angiography".ti,ab OR "Magnetic Resonance Angiographies".ti,ab OR "MRA".ti,ab OR *"perfusion weighted imaging"/ OR "Perfusion Magnetic Resonance Imaging".ti,ab OR "Perfusion Weighted MRI".ti,ab OR *"Computed Tomographic Angiography"/ OR "Computed Tomography Angiography".ti,ab OR "Computed Tomography Angiographies".ti,ab OR "CT Angiography".ti,ab OR "CT Angiographies".ti,ab OR "CTA".ti,ab OR *"duplex Doppler ultrasonography"/ OR "Duplex Doppler".ti,ab OR "Doppler Duplex".ti,ab OR *"color Doppler flowmetry"/ OR "Doppler Color".ti,ab OR "Color Doppler".ti,ab OR "Doppler Colour".ti,ab OR "Colour Doppler".ti,ab OR "color duplex".ti,ab OR "colour duplex".ti,ab OR "duplex color".ti,ab OR "duplex colour".ti,ab OR "CDUS".ti,ab OR exp *"Plethysmography"/ OR "Plethysmography".ti,ab OR "Plethysmogra*".ti,ab OR "color spectral waveforms".ti,ab OR "color spectral waveform".ti,ab OR "color spectral wave forms".ti,ab OR "color spectral wave form".ti,ab OR "colour spectral waveforms".ti,ab OR "colour spectral waveform".ti,ab OR "colour spectral wave forms".ti,ab OR "colour spectral wave form".ti,ab))) AND english.la | 589  160 meeting abstracts | 250  156 meeting abstracts |
|  |  |  |  |
| Web of Science | ((ti=("Peripheral Occlusive Arterial Disease" OR "Peripheral Arterial Disease" OR "Peripheral Arterial Diseases" OR "Peripheral Artery Disease" OR "Peripheral Artery Diseases" OR "PAD" OR "peripheral arterial occlusive disease" OR "peripheral arterial occlusive diseases" OR "peripheral artery occlusive disease" OR "peripheral artery occlusive diseases" OR "Peripheral Vascular Disease" OR "Peripheral Angiopathies" OR "Peripheral Angiopathy" OR "Peripheral Arteriopathies" OR "Peripheral Arteriopathy" OR "Peripheral Vasculopathies" OR "Peripheral Vasculopathy" OR "Monckeberg Medial Calcific Sclerosis" OR "Monckeberg's Medial Calcific Sclerosis" OR "Monckeberg's Sclerosis" OR "Monckeberg Sclerosis" OR "Monckebergs Sclerosis" OR "Mönckeberg's Medial Calcific Sclerosis" OR "Mönckeberg's Sclerosis" OR "Mönckeberg Sclerosis" OR "Mönckebergs Sclerosis" OR "Monckeberg" OR "Monckeberg*" OR "Moenckeberg" OR "Moenckeberg*" OR "Medial Calcific Sclerosis" OR "Medial Calcific Scleroses" OR "Mönckeberg Medial Calcific Sclerosis" OR "incompressible arter*" OR "incompressible vessel*" OR "medial calcified arter*" OR (("Peripheral Occlusive Arterial Disease" OR "Peripheral Vascular Disease") AND ("peripheral" OR "peripheral*" OR "limbs" OR "limb" OR "leg" OR "legs" OR "extremity" OR "extremities" OR "arm" OR "arms")) OR "Intermittent Claudication" OR "Intermittent Claudication" OR "Leriche Syndrome" OR "Leriche Syndrome" OR "Leriche's Syndrome" OR "Leriches Syndrome" OR "Arteriosclerosis Obliterans" OR (("Atherosclerosis" OR "Arteriosclerosis" OR "Atherosclerosis" OR "Arteriosclerosis") AND ("peripheral" OR "peripheral*" OR "limbs" OR "limb" OR "leg" OR "legs" OR "extremity" OR "extremities" OR "arm" OR "arms"))) AND ti=("Ankle Brachial Index" OR "Ankle Brachial Indices" OR "Ankle-Brachial Index" OR "Ankle-Brachial Indices" OR "anklebrachial index" OR "anklebrachialindex" OR "ankle brachial" OR "anklebrachial" OR "ABI" OR "Toe Brachial Index" OR "Toe Brachial Indices" OR "Toe-Brachial Index" OR "Toe-Brachial Indices" OR "Toe Pressure" OR "toe brachial" OR "toebrachial" OR "TBI" OR "TP" OR "Oximetry" OR "Oximetry" OR "Oximetries" OR "Oximetr*" OR "Pulse Oximetries" OR "Pulse Oximetry" OR "transcutaneous oxygen tension" OR "transcutaneous oxygen" OR "Pulse" OR "Pulse*" OR "pulsation" OR "pulsations" OR "Transcutaneous Oxygen Monitoring" OR "Transcutaneous Blood Gas Monitoring" OR "Transcutaneous Capnometry" OR "PtcO2" OR "TcPCO2" OR "Doppler waveform" OR "Doppler waveforms" OR "Doppler wave form" OR "Doppler wave forms" OR "pulsed Doppler echocardiography" OR "Pulsed Doppler" OR "Doppler Pulsed" OR "non-invasive" OR "non-invasiv*" OR "point-of-care test" OR "point-of-care tests") AND ti=("Angiography" OR "Angiography" OR "Angiographies" OR "Angiogram" OR "Angiograms" OR "Angiogra*" OR "Arteriography" OR "Arteriographies" OR "Arteriogra*" OR "Digital Subtraction Angiography" OR "DSA" OR "Magnetic Resonance Angiography" OR "MRI Angiography" OR "MRI Angiographies" OR "Magnetic Resonance Angiography" OR "Magnetic Resonance Angiographies" OR "MRA" OR "perfusion weighted imaging" OR "Perfusion Magnetic Resonance Imaging" OR "Perfusion Weighted MRI" OR "Computed Tomographic Angiography" OR "Computed Tomography Angiography" OR "Computed Tomography Angiographies" OR "CT Angiography" OR "CT Angiographies" OR "CTA" OR "duplex Doppler ultrasonography" OR "Duplex Doppler" OR "Doppler Duplex" OR "color Doppler flowmetry" OR "Doppler Color" OR "Color Doppler" OR "Doppler Colour" OR "Colour Doppler" OR "color duplex" OR "colour duplex" OR "duplex color" OR "duplex colour" OR "CDUS" OR "Plethysmography" OR "Plethysmography" OR "Plethysmogra*" OR "color spectral waveforms" OR "color spectral waveform" OR "color spectral wave forms" OR "color spectral wave form" OR "colour spectral waveforms" OR "colour spectral waveform" OR "colour spectral wave forms" OR "colour spectral wave form") AND TS=("Sensitivity and Specificity" OR "Sensitivity" OR "Specificity" OR "Predictive Value" OR "ROC Curve" OR "Signal-To-Noise" OR "limit of detection" OR "Cohort Analysis" OR "Follow Up" OR "Longitudinal Study" OR "Prospective Study" OR "Retrospective Study" OR "Cohort" OR "Cohorts" OR "Follow-Up" OR "Longitudinal" OR "Prospective" OR "Retrospective" OR "Cross-Sectional Study" OR "Cross-Sectional" OR "Observational Study" OR "Observational Study" OR "effectiveness" OR "efficacy")) OR (ti=("Peripheral Occlusive Arterial Disease" OR "Peripheral Arterial Disease" OR "Peripheral Arterial Diseases" OR "Peripheral Artery Disease" OR "Peripheral Artery Diseases" OR "PAD" OR "peripheral arterial occlusive disease" OR "peripheral arterial occlusive diseases" OR "peripheral artery occlusive disease" OR "peripheral artery occlusive diseases" OR "Peripheral Vascular Disease" OR "Peripheral Angiopathies" OR "Peripheral Angiopathy" OR "Peripheral Arteriopathies" OR "Peripheral Arteriopathy" OR "Peripheral Vasculopathies" OR "Peripheral Vasculopathy" OR "Monckeberg Medial Calcific Sclerosis" OR "Monckeberg's Medial Calcific Sclerosis" OR "Monckeberg's Sclerosis" OR "Monckeberg Sclerosis" OR "Monckebergs Sclerosis" OR "Mönckeberg's Medial Calcific Sclerosis" OR "Mönckeberg's Sclerosis" OR "Mönckeberg Sclerosis" OR "Mönckebergs Sclerosis" OR "Monckeberg" OR "Monckeberg*" OR "Moenckeberg" OR "Moenckeberg*" OR "Medial Calcific Sclerosis" OR "Medial Calcific Scleroses" OR "Mönckeberg Medial Calcific Sclerosis" OR "incompressible arter*" OR "incompressible vessel*" OR "medial calcified arter*" OR (("Peripheral Occlusive Arterial Disease" OR "Peripheral Vascular Disease") AND ("peripheral" OR "peripheral*" OR "limbs" OR "limb" OR "leg" OR "legs" OR "extremity" OR "extremities" OR "arm" OR "arms")) OR "Intermittent Claudication" OR "Intermittent Claudication" OR "Leriche Syndrome" OR "Leriche Syndrome" OR "Leriche's Syndrome" OR "Leriches Syndrome" OR "Arteriosclerosis Obliterans" OR (("Atherosclerosis" OR "Arteriosclerosis" OR "Atherosclerosis" OR "Arteriosclerosis") AND ("peripheral" OR "peripheral*" OR "limbs" OR "limb" OR "leg" OR "legs" OR "extremity" OR "extremities" OR "arm" OR "arms"))) AND ti=("Ankle Brachial Index" OR "Ankle Brachial Indices" OR "Ankle-Brachial Index" OR "Ankle-Brachial Indices" OR "anklebrachial index" OR "anklebrachialindex" OR "ankle brachial" OR "anklebrachial" OR "ABI" OR "Toe Brachial Index" OR "Toe Brachial Indices" OR "Toe-Brachial Index" OR "Toe-Brachial Indices" OR "Toe Pressure" OR "toe brachial" OR "toebrachial" OR "TBI" OR "TP" OR "Oximetry" OR "Oximetry" OR "Oximetries" OR "Oximetr*" OR "Pulse Oximetries" OR "Pulse Oximetry" OR "transcutaneous oxygen tension" OR "transcutaneous oxygen" OR "Pulse" OR "Pulse*" OR "pulsation" OR "pulsations" OR "Transcutaneous Oxygen Monitoring" OR "Transcutaneous Blood Gas Monitoring" OR "Transcutaneous Capnometry" OR "PtcO2" OR "TcPCO2" OR "Doppler waveform" OR "Doppler waveforms" OR "Doppler wave form" OR "Doppler wave forms" OR "pulsed Doppler echocardiography" OR "Pulsed Doppler" OR "Doppler Pulsed" OR "non-invasive" OR "non-invasiv*" OR "point-of-care test" OR "point-of-care tests") AND ti=("Sensitivity and Specificity" OR "Sensitivity" OR "Specificity" OR "Predictive Value" OR "ROC Curve" OR "Signal-To-Noise" OR "limit of detection" OR "Cohort Analysis" OR "Follow Up" OR "Longitudinal Study" OR "Prospective Study" OR "Retrospective Study" OR "Cohort" OR "Cohorts" OR "Follow-Up" OR "Longitudinal" OR "Prospective" OR "Retrospective" OR "Cross-Sectional Study" OR "Cross-Sectional" OR "Observational Study" OR "Observational Study" OR "effectiveness" OR "efficacy")) OR (ti=("Peripheral Occlusive Arterial Disease" OR "Peripheral Arterial Disease" OR "Peripheral Arterial Diseases" OR "Peripheral Artery Disease" OR "Peripheral Artery Diseases" OR "PAD" OR "peripheral arterial occlusive disease" OR "peripheral arterial occlusive diseases" OR "peripheral artery occlusive disease" OR "peripheral artery occlusive diseases" OR "Peripheral Vascular Disease" OR "Peripheral Angiopathies" OR "Peripheral Angiopathy" OR "Peripheral Arteriopathies" OR "Peripheral Arteriopathy" OR "Peripheral Vasculopathies" OR "Peripheral Vasculopathy" OR "Monckeberg Medial Calcific Sclerosis" OR "Monckeberg's Medial Calcific Sclerosis" OR "Monckeberg's Sclerosis" OR "Monckeberg Sclerosis" OR "Monckebergs Sclerosis" OR "Mönckeberg's Medial Calcific Sclerosis" OR "Mönckeberg's Sclerosis" OR "Mönckeberg Sclerosis" OR "Mönckebergs Sclerosis" OR "Monckeberg" OR "Monckeberg*" OR "Moenckeberg" OR "Moenckeberg*" OR "Medial Calcific Sclerosis" OR "Medial Calcific Scleroses" OR "Mönckeberg Medial Calcific Sclerosis" OR "incompressible arter*" OR "incompressible vessel*" OR "medial calcified arter*" OR (("Peripheral Occlusive Arterial Disease" OR "Peripheral Vascular Disease") AND ("peripheral" OR "peripheral*" OR "limbs" OR "limb" OR "leg" OR "legs" OR "extremity" OR "extremities" OR "arm" OR "arms")) OR "Intermittent Claudication" OR "Intermittent Claudication" OR "Leriche Syndrome" OR "Leriche Syndrome" OR "Leriche's Syndrome" OR "Leriches Syndrome" OR "Arteriosclerosis Obliterans" OR (("Atherosclerosis" OR "Arteriosclerosis" OR "Atherosclerosis" OR "Arteriosclerosis") AND ("peripheral" OR "peripheral*" OR "limbs" OR "limb" OR "leg" OR "legs" OR "extremity" OR "extremities" OR "arm" OR "arms"))) AND ti=("Ankle Brachial Index" OR "Ankle Brachial Indices" OR "Ankle-Brachial Index" OR "Ankle-Brachial Indices" OR "anklebrachial index" OR "anklebrachialindex" OR "ankle brachial" OR "anklebrachial" OR "ABI" OR "Toe Brachial Index" OR "Toe Brachial Indices" OR "Toe-Brachial Index" OR "Toe-Brachial Indices" OR "Toe Pressure" OR "toe brachial" OR "toebrachial" OR "TBI" OR "TP" OR "Oximetry" OR "Oximetry" OR "Oximetries" OR "Oximetr*" OR "Pulse Oximetries" OR "Pulse Oximetry" OR "transcutaneous oxygen tension" OR "transcutaneous oxygen" OR "Pulse" OR "Pulse*" OR "pulsation" OR "pulsations" OR "Transcutaneous Oxygen Monitoring" OR "Transcutaneous Blood Gas Monitoring" OR "Transcutaneous Capnometry" OR "PtcO2" OR "TcPCO2" OR "Doppler waveform" OR "Doppler waveforms" OR "Doppler wave form" OR "Doppler wave forms" OR "pulsed Doppler echocardiography" OR "Pulsed Doppler" OR "Doppler Pulsed" OR "non-invasive" OR "non-invasiv*" OR "point-of-care test" OR "point-of-care tests" OR "point-of-care test" OR "point-of-care tests" OR "point-of-care test" OR "point-of-care tests") AND ti=("Angiography" OR "Angiography" OR "Angiographies" OR "Angiogram" OR "Angiograms" OR "Angiogra*" OR "Arteriography" OR "Arteriographies" OR "Arteriogra*" OR "Digital Subtraction Angiography" OR "DSA" OR "Magnetic Resonance Angiography" OR "MRI Angiography" OR "MRI Angiographies" OR "Magnetic Resonance Angiography" OR "Magnetic Resonance Angiographies" OR "MRA" OR "perfusion weighted imaging" OR "Perfusion Magnetic Resonance Imaging" OR "Perfusion Weighted MRI" OR "Computed Tomographic Angiography" OR "Computed Tomography Angiography" OR "Computed Tomography Angiographies" OR "CT Angiography" OR "CT Angiographies" OR "CTA" OR "duplex Doppler ultrasonography" OR "Duplex Doppler" OR "Doppler Duplex" OR "color Doppler flowmetry" OR "Doppler Color" OR "Color Doppler" OR "Doppler Colour" OR "Colour Doppler" OR "color duplex" OR "colour duplex" OR "duplex color" OR "duplex colour" OR "CDUS" OR "Plethysmography" OR "Plethysmography" OR "Plethysmogra*" OR "color spectral waveforms" OR "color spectral waveform" OR "color spectral wave forms" OR "color spectral wave form" OR "colour spectral waveforms" OR "colour spectral waveform" OR "colour spectral wave forms" OR "colour spectral wave form") AND TS=("Monckeberg Medial Calcific Sclerosis" OR "Monckeberg's Medial Calcific Sclerosis".mp OR "Monckeberg's Sclerosis".mp OR "Monckeberg Sclerosis".mp OR "Monckebergs Sclerosis".mp OR "Mönckeberg's Medial Calcific Sclerosis".mp OR "Mönckeberg's Sclerosis".mp OR "Mönckeberg Sclerosis".mp OR "Mönckebergs Sclerosis".mp OR "Monckeberg".mp OR "Monckeberg*".mp OR "Moenckeberg".mp OR "Moenckeberg*".mp OR "Medial Calcific Sclerosis".mp OR "Medial Calcific Scleroses".mp OR "Mönckeberg Medial Calcific Sclerosis".mp OR "incompressible arter*".mp OR "incompressible vessel*".mp OR "medial calcified arter*".mp OR "media sclerosis".mp OR "mediasclerosis".mp OR "in-compressible artery".mp OR "incompressible artery".mp OR "in-compressible arteries".mp OR "incompressible arteries".mp OR "in-compressible vessel".mp OR "incompressible vessel".mp OR "in-compressible vessels".mp OR "incompressible vessels".mp OR "non-compressible artery".mp OR "noncompressible artery".mp OR "non-compressible arteries".mp OR "noncompressible arteries".mp OR "non-compressible vessel".mp OR "noncompressible vessel".mp OR "non-compressible vessels".mp OR "noncompressible vessels".mp OR "in compress*".mp OR "incompress*".mp OR "non compress*".mp OR "noncompress*".mp OR "calcifications".mp OR "calcification".mp OR "calcificat*".mp OR "Calcification")) OR (ti=("Peripheral Occlusive Arterial Disease" OR "Peripheral Arterial Disease" OR "Peripheral Arterial Diseases" OR "Peripheral Artery Disease" OR "Peripheral Artery Diseases" OR "PAD" OR "peripheral arterial occlusive disease" OR "peripheral arterial occlusive diseases" OR "peripheral artery occlusive disease" OR "peripheral artery occlusive diseases" OR "Peripheral Vascular Disease" OR "Peripheral Angiopathies" OR "Peripheral Angiopathy" OR "Peripheral Arteriopathies" OR "Peripheral Arteriopathy" OR "Peripheral Vasculopathies" OR "Peripheral Vasculopathy" OR "Monckeberg Medial Calcific Sclerosis" OR "Monckeberg's Medial Calcific Sclerosis" OR "Monckeberg's Sclerosis" OR "Monckeberg Sclerosis" OR "Monckebergs Sclerosis" OR "Mönckeberg's Medial Calcific Sclerosis" OR "Mönckeberg's Sclerosis" OR "Mönckeberg Sclerosis" OR "Mönckebergs Sclerosis" OR "Monckeberg" OR "Monckeberg*" OR "Moenckeberg" OR "Moenckeberg*" OR "Medial Calcific Sclerosis" OR "Medial Calcific Scleroses" OR "Mönckeberg Medial Calcific Sclerosis" OR "incompressible arter*" OR "incompressible vessel*" OR "medial calcified arter*" OR (("Peripheral Occlusive Arterial Disease" OR "Peripheral Vascular Disease") AND ("peripheral" OR "peripheral*" OR "limbs" OR "limb" OR "leg" OR "legs" OR "extremity" OR "extremities" OR "arm" OR "arms")) OR "Intermittent Claudication" OR "Intermittent Claudication" OR "Leriche Syndrome" OR "Leriche Syndrome" OR "Leriche's Syndrome" OR "Leriches Syndrome" OR "Arteriosclerosis Obliterans" OR (("Atherosclerosis" OR "Arteriosclerosis" OR "Atherosclerosis" OR "Arteriosclerosis") AND ("peripheral" OR "peripheral*" OR "limbs" OR "limb" OR "leg" OR "legs" OR "extremity" OR "extremities" OR "arm" OR "arms"))) AND ti=("clinical test" OR "clinical tests" OR "screening test" OR "screening tests" OR "point-of-care test" OR "point-of-care tests")) OR (ti=("Ankle Brachial Index" OR "Ankle Brachial Indices" OR "Ankle-Brachial Index" OR "Ankle-Brachial Indices" OR "anklebrachial index" OR "anklebrachialindex" OR "ankle brachial" OR "anklebrachial" OR "ABI" OR "Toe Brachial Index" OR "Toe Brachial Indices" OR "Toe-Brachial Index" OR "Toe-Brachial Indices" OR "Toe Pressure" OR "toe brachial" OR "toebrachial" OR "TBI" OR "TP" OR "Oximetry" OR "Oximetry" OR "Oximetries" OR "Oximetr*" OR "Pulse Oximetries" OR "Pulse Oximetry" OR "transcutaneous oxygen tension" OR "transcutaneous oxygen" OR "Pulse" OR "Pulse*" OR "pulsation" OR "pulsations" OR "Transcutaneous Oxygen Monitoring" OR "Transcutaneous Blood Gas Monitoring" OR "Transcutaneous Capnometry" OR "PtcO2" OR "TcPCO2" OR "Doppler waveform" OR "Doppler waveforms" OR "Doppler wave form" OR "Doppler wave forms" OR "pulsed Doppler echocardiography" OR "Pulsed Doppler" OR "Doppler Pulsed" OR "non-invasive" OR "non-invasiv*" OR "point-of-care test" OR "point-of-care tests" OR "point-of-care test" OR "point-of-care tests") AND ti=("Angiography" OR "Angiography" OR "Angiographies" OR "Angiogram" OR "Angiograms" OR "Angiogra*" OR "Arteriography" OR "Arteriographies" OR "Arteriogra*" OR "Digital Subtraction Angiography" OR "DSA" OR "Magnetic Resonance Angiography" OR "MRI Angiography" OR "MRI Angiographies" OR "Magnetic Resonance Angiography" OR "Magnetic Resonance Angiographies" OR "MRA" OR "perfusion weighted imaging" OR "Perfusion Magnetic Resonance Imaging" OR "Perfusion Weighted MRI" OR "Computed Tomographic Angiography" OR "Computed Tomography Angiography" OR "Computed Tomography Angiographies" OR "CT Angiography" OR "CT Angiographies" OR "CTA" OR "duplex Doppler ultrasonography" OR "Duplex Doppler" OR "Doppler Duplex" OR "color Doppler flowmetry" OR "Doppler Color" OR "Color Doppler" OR "Doppler Colour" OR "Colour Doppler" OR "color duplex" OR "colour duplex" OR "duplex color" OR "duplex colour" OR "CDUS" OR "Plethysmography" OR "Plethysmography" OR "Plethysmogra*" OR "color spectral waveforms" OR "color spectral waveform" OR "color spectral wave forms" OR "color spectral wave form" OR "colour spectral waveforms" OR "colour spectral waveform" OR "colour spectral wave forms" OR "colour spectral wave form") AND ti=("Diabetes Mellitus" OR "diabetes" OR "diabet*" OR "Chronic Kidney Failure" OR "end stage renal disease" OR "Chronic Kidney Disease" OR "Chronic Kidney Diseases" OR "Chronic Kidney Failure" OR "Chronic Kidney Insufficiency" OR "Chronic Renal Disease" OR "Chronic Renal Diseases" OR "Chronic Renal Failure" OR "Chronic Renal Insufficiency" OR "End Stage Kidney Disease" OR "End Stage Renal Disease" OR "End-Stage Renal Failure" OR "End-Stage Kidney Failure" OR "ESRD")) OR (ti=("Peripheral Occlusive Arterial Disease" OR "Peripheral Arterial Disease" OR "Peripheral Arterial Diseases" OR "Peripheral Artery Disease" OR "Peripheral Artery Diseases" OR "PAD" OR "peripheral arterial occlusive disease" OR "peripheral arterial occlusive diseases" OR "peripheral artery occlusive disease" OR "peripheral artery occlusive diseases" OR "Peripheral Vascular Disease" OR "Peripheral Angiopathies" OR "Peripheral Angiopathy" OR "Peripheral Arteriopathies" OR "Peripheral Arteriopathy" OR "Peripheral Vasculopathies" OR "Peripheral Vasculopathy" OR "Monckeberg Medial Calcific Sclerosis" OR "Monckeberg's Medial Calcific Sclerosis" OR "Monckeberg's Sclerosis" OR "Monckeberg Sclerosis" OR "Monckebergs Sclerosis" OR "Mönckeberg's Medial Calcific Sclerosis" OR "Mönckeberg's Sclerosis" OR "Mönckeberg Sclerosis" OR "Mönckebergs Sclerosis" OR "Monckeberg" OR "Monckeberg*" OR "Moenckeberg" OR "Moenckeberg*" OR "Medial Calcific Sclerosis" OR "Medial Calcific Scleroses" OR "Mönckeberg Medial Calcific Sclerosis" OR "incompressible arter*" OR "incompressible vessel*" OR "medial calcified arter*" OR (("Peripheral Occlusive Arterial Disease" OR "Peripheral Vascular Disease") AND ("peripheral" OR "peripheral*" OR "limbs" OR "limb" OR "leg" OR "legs" OR "extremity" OR "extremities" OR "arm" OR "arms")) OR "Intermittent Claudication" OR "Intermittent Claudication" OR "Leriche Syndrome" OR "Leriche Syndrome" OR "Leriche's Syndrome" OR "Leriches Syndrome" OR "Arteriosclerosis Obliterans" OR (("Atherosclerosis" OR "Arteriosclerosis" OR "Atherosclerosis" OR "Arteriosclerosis") AND ("peripheral" OR "peripheral*" OR "limbs" OR "limb" OR "leg" OR "legs" OR "extremity" OR "extremities" OR "arm" OR "arms"))) AND ti=("Diabetes Mellitus" OR "diabetes" OR "diabet*" OR "Chronic Kidney Failure" OR "end stage renal disease" OR "Chronic Kidney Disease" OR "Chronic Kidney Diseases" OR "Chronic Kidney Failure" OR "Chronic Kidney Insufficiency" OR "Chronic Renal Disease" OR "Chronic Renal Diseases" OR "Chronic Renal Failure" OR "Chronic Renal Insufficiency" OR "End Stage Kidney Disease" OR "End Stage Renal Disease" OR "End-Stage Renal Failure" OR "End-Stage Kidney Failure" OR "ESRD") AND ts=("Ankle Brachial Index" OR "Ankle Brachial Indices" OR "Ankle-Brachial Index" OR "Ankle-Brachial Indices" OR "anklebrachial index" OR "anklebrachialindex" OR "ankle brachial" OR "anklebrachial" OR "ABI" OR "Toe Brachial Index" OR "Toe Brachial Indices" OR "Toe-Brachial Index" OR "Toe-Brachial Indices" OR "Toe Pressure" OR "toe brachial" OR "toebrachial" OR "TBI" OR "TP" OR "Oximetry" OR "Oximetry" OR "Oximetries" OR "Oximetr*" OR "Pulse Oximetries" OR "Pulse Oximetry" OR "transcutaneous oxygen tension" OR "transcutaneous oxygen" OR "Pulse" OR "Pulse*" OR "pulsation" OR "pulsations" OR "Transcutaneous Oxygen Monitoring" OR "Transcutaneous Blood Gas Monitoring" OR "Transcutaneous Capnometry" OR "PtcO2" OR "TcPCO2" OR "Doppler waveform" OR "Doppler waveforms" OR "Doppler wave form" OR "Doppler wave forms" OR "pulsed Doppler echocardiography" OR "Pulsed Doppler" OR "Doppler Pulsed" OR "non-invasive" OR "non-invasiv*" OR "point-of-care test" OR "point-of-care tests" OR "point-of-care test" OR "point-of-care tests") AND ts=("Angiography" OR "Angiography" OR "Angiographies" OR "Angiogram" OR "Angiograms" OR "Angiogra*" OR "Arteriography" OR "Arteriographies" OR "Arteriogra*" OR "Digital Subtraction Angiography" OR "DSA" OR "Magnetic Resonance Angiography" OR "MRI Angiography" OR "MRI Angiographies" OR "Magnetic Resonance Angiography" OR "Magnetic Resonance Angiographies" OR "MRA" OR "perfusion weighted imaging" OR "Perfusion Magnetic Resonance Imaging" OR "Perfusion Weighted MRI" OR "Computed Tomographic Angiography" OR "Computed Tomography Angiography" OR "Computed Tomography Angiographies" OR "CT Angiography" OR "CT Angiographies" OR "CTA" OR "duplex Doppler ultrasonography" OR "Duplex Doppler" OR "Doppler Duplex" OR "color Doppler flowmetry" OR "Doppler Color" OR "Color Doppler" OR "Doppler Colour" OR "Colour Doppler" OR "color duplex" OR "colour duplex" OR "duplex color" OR "duplex colour" OR "CDUS" OR "Plethysmography" OR "Plethysmography" OR "Plethysmogra*" OR "color spectral waveforms" OR "color spectral waveform" OR "color spectral wave forms" OR "color spectral wave form" OR "colour spectral waveforms" OR "colour spectral waveform" OR "colour spectral wave forms" OR "colour spectral wave form"))) AND la=english | 91  18 meeting abstracts | 15  8 meeting abstracts |
|  |  |  |  |
| Cochrane Library | (("Peripheral Occlusive Arterial Disease" OR "Peripheral Arterial Disease" OR "Peripheral Arterial Diseases" OR "Peripheral Artery Disease" OR "Peripheral Artery Diseases" OR "PAD" OR "peripheral arterial occlusive disease" OR "peripheral arterial occlusive diseases" OR "peripheral artery occlusive disease" OR "peripheral artery occlusive diseases" OR "Peripheral Vascular Disease" OR "Peripheral Angiopathies" OR "Peripheral Angiopathy" OR "Peripheral Arteriopathies" OR "Peripheral Arteriopathy" OR "Peripheral Vasculopathies" OR "Peripheral Vasculopathy" OR "Monckeberg Medial Calcific Sclerosis" OR "Monckeberg's Medial Calcific Sclerosis" OR "Monckeberg's Sclerosis" OR "Monckeberg Sclerosis" OR "Monckebergs Sclerosis" OR "Mönckeberg's Medial Calcific Sclerosis" OR "Mönckeberg's Sclerosis" OR "Mönckeberg Sclerosis" OR "Mönckebergs Sclerosis" OR "Monckeberg" OR "Monckeberg*" OR "Moenckeberg" OR "Moenckeberg*" OR "Medial Calcific Sclerosis" OR "Medial Calcific Scleroses" OR "Mönckeberg Medial Calcific Sclerosis" OR "incompressible arter*" OR "incompressible vessel*" OR "medial calcified arter*" OR (("Peripheral Occlusive Arterial Disease" OR "Peripheral Vascular Disease") AND ("peripheral" OR "peripheral*" OR "limbs" OR "limb" OR "leg" OR "legs" OR "extremity" OR "extremities" OR "arm" OR "arms")) OR "Intermittent Claudication" OR "Intermittent Claudication" OR "Leriche Syndrome" OR "Leriche Syndrome" OR "Leriche's Syndrome" OR "Leriches Syndrome" OR "Arteriosclerosis Obliterans" OR (("Atherosclerosis" OR "Arteriosclerosis" OR "Atherosclerosis" OR "Arteriosclerosis") AND ("peripheral" OR "peripheral*" OR "limbs" OR "limb" OR "leg" OR "legs" OR "extremity" OR "extremities" OR "arm" OR "arms"))):ti AND ("Ankle Brachial Index" OR "Ankle Brachial Indices" OR "Ankle Brachial Index" OR "Ankle Brachial Indices" OR "anklebrachial index" OR "anklebrachialindex" OR "ankle brachial" OR "anklebrachial" OR "ABI" OR "Toe Brachial Index" OR "Toe Brachial Indices" OR "Toe Brachial Index" OR "Toe Brachial Indices" OR "Toe Pressure" OR "toe brachial" OR "toebrachial" OR "TBI" OR "TP" OR "Oximetry" OR "Oximetry" OR "Oximetries" OR "Oximetr*" OR "Pulse Oximetries" OR "Pulse Oximetry" OR "transcutaneous oxygen tension" OR "transcutaneous oxygen" OR "Pulse" OR "Pulse*" OR "pulsation" OR "pulsations" OR "Transcutaneous Oxygen Monitoring" OR "Transcutaneous Blood Gas Monitoring" OR "Transcutaneous Capnometry" OR "PtcO2" OR "TcPCO2" OR "Doppler waveform" OR "Doppler waveforms" OR "Doppler wave form" OR "Doppler wave forms" OR "pulsed Doppler echocardiography" OR "Pulsed Doppler" OR "Doppler Pulsed" OR "non invasive" OR "non invasiv*" OR "point of care test" OR "point of care tests"):ti AND ("Angiography" OR "Angiography" OR "Angiographies" OR "Angiogram" OR "Angiograms" OR "Angiogra*" OR "Arteriography" OR "Arteriographies" OR "Arteriogra*" OR "Digital Subtraction Angiography" OR "DSA" OR "Magnetic Resonance Angiography" OR "MRI Angiography" OR "MRI Angiographies" OR "Magnetic Resonance Angiography" OR "Magnetic Resonance Angiographies" OR "MRA" OR "perfusion weighted imaging" OR "Perfusion Magnetic Resonance Imaging" OR "Perfusion Weighted MRI" OR "Computed Tomographic Angiography" OR "Computed Tomography Angiography" OR "Computed Tomography Angiographies" OR "CT Angiography" OR "CT Angiographies" OR "CTA" OR "duplex Doppler ultrasonography" OR "Duplex Doppler" OR "Doppler Duplex" OR "color Doppler flowmetry" OR "Doppler Color" OR "Color Doppler" OR "Doppler Colour" OR "Colour Doppler" OR "color duplex" OR "colour duplex" OR "duplex color" OR "duplex colour" OR "CDUS" OR "Plethysmography" OR "Plethysmography" OR "Plethysmogra*" OR "color spectral waveforms" OR "color spectral waveform" OR "color spectral wave forms" OR "color spectral wave form" OR "colour spectral waveforms" OR "colour spectral waveform" OR "colour spectral wave forms" OR "colour spectral wave form"):ti,ab,kw) OR (("Peripheral Occlusive Arterial Disease" OR "Peripheral Arterial Disease" OR "Peripheral Arterial Diseases" OR "Peripheral Artery Disease" OR "Peripheral Artery Diseases" OR "PAD" OR "peripheral arterial occlusive disease" OR "peripheral arterial occlusive diseases" OR "peripheral artery occlusive disease" OR "peripheral artery occlusive diseases" OR "Peripheral Vascular Disease" OR "Peripheral Angiopathies" OR "Peripheral Angiopathy" OR "Peripheral Arteriopathies" OR "Peripheral Arteriopathy" OR "Peripheral Vasculopathies" OR "Peripheral Vasculopathy" OR "Monckeberg Medial Calcific Sclerosis" OR "Monckeberg's Medial Calcific Sclerosis" OR "Monckeberg's Sclerosis" OR "Monckeberg Sclerosis" OR "Monckebergs Sclerosis" OR "Mönckeberg's Medial Calcific Sclerosis" OR "Mönckeberg's Sclerosis" OR "Mönckeberg Sclerosis" OR "Mönckebergs Sclerosis" OR "Monckeberg" OR "Monckeberg*" OR "Moenckeberg" OR "Moenckeberg*" OR "Medial Calcific Sclerosis" OR "Medial Calcific Scleroses" OR "Mönckeberg Medial Calcific Sclerosis" OR "incompressible arter*" OR "incompressible vessel*" OR "medial calcified arter*" OR (("Peripheral Occlusive Arterial Disease" OR "Peripheral Vascular Disease") AND ("peripheral" OR "peripheral*" OR "limbs" OR "limb" OR "leg" OR "legs" OR "extremity" OR "extremities" OR "arm" OR "arms")) OR "Intermittent Claudication" OR "Intermittent Claudication" OR "Leriche Syndrome" OR "Leriche Syndrome" OR "Leriche's Syndrome" OR "Leriches Syndrome" OR "Arteriosclerosis Obliterans" OR (("Atherosclerosis" OR "Arteriosclerosis" OR "Atherosclerosis" OR "Arteriosclerosis") AND ("peripheral" OR "peripheral*" OR "limbs" OR "limb" OR "leg" OR "legs" OR "extremity" OR "extremities" OR "arm" OR "arms"))):ti AND ("Ankle Brachial Index" OR "Ankle Brachial Indices" OR "Ankle Brachial Index" OR "Ankle Brachial Indices" OR "anklebrachial index" OR "anklebrachialindex" OR "ankle brachial" OR "anklebrachial" OR "ABI" OR "Toe Brachial Index" OR "Toe Brachial Indices" OR "Toe Brachial Index" OR "Toe Brachial Indices" OR "Toe Pressure" OR "toe brachial" OR "toebrachial" OR "TBI" OR "TP" OR "Oximetry" OR "Oximetry" OR "Oximetries" OR "Oximetr*" OR "Pulse Oximetries" OR "Pulse Oximetry" OR "transcutaneous oxygen tension" OR "transcutaneous oxygen" OR "Pulse" OR "Pulse*" OR "pulsation" OR "pulsations" OR "Transcutaneous Oxygen Monitoring" OR "Transcutaneous Blood Gas Monitoring" OR "Transcutaneous Capnometry" OR "PtcO2" OR "TcPCO2" OR "Doppler waveform" OR "Doppler waveforms" OR "Doppler wave form" OR "Doppler wave forms" OR "pulsed Doppler echocardiography" OR "Pulsed Doppler" OR "Doppler Pulsed" OR "non invasive" OR "non invasiv*" OR "point of care test" OR "point of care tests"):ti,ab,kw AND ("Angiography" OR "Angiography" OR "Angiographies" OR "Angiogram" OR "Angiograms" OR "Angiogra*" OR "Arteriography" OR "Arteriographies" OR "Arteriogra*" OR "Digital Subtraction Angiography" OR "DSA" OR "Magnetic Resonance Angiography" OR "MRI Angiography" OR "MRI Angiographies" OR "Magnetic Resonance Angiography" OR "Magnetic Resonance Angiographies" OR "MRA" OR "perfusion weighted imaging" OR "Perfusion Magnetic Resonance Imaging" OR "Perfusion Weighted MRI" OR "Computed Tomographic Angiography" OR "Computed Tomography Angiography" OR "Computed Tomography Angiographies" OR "CT Angiography" OR "CT Angiographies" OR "CTA" OR "duplex Doppler ultrasonography" OR "Duplex Doppler" OR "Doppler Duplex" OR "color Doppler flowmetry" OR "Doppler Color" OR "Color Doppler" OR "Doppler Colour" OR "Colour Doppler" OR "color duplex" OR "colour duplex" OR "duplex color" OR "duplex colour" OR "CDUS" OR "Plethysmography" OR "Plethysmography" OR "Plethysmogra*" OR "color spectral waveforms" OR "color spectral waveform" OR "color spectral wave forms" OR "color spectral wave form" OR "colour spectral waveforms" OR "colour spectral waveform" OR "colour spectral wave forms" OR "colour spectral wave form"):ti) OR (("Peripheral Occlusive Arterial Disease" OR "Peripheral Arterial Disease" OR "Peripheral Arterial Diseases" OR "Peripheral Artery Disease" OR "Peripheral Artery Diseases" OR "PAD" OR "peripheral arterial occlusive disease" OR "peripheral arterial occlusive diseases" OR "peripheral artery occlusive disease" OR "peripheral artery occlusive diseases" OR "Peripheral Vascular Disease" OR "Peripheral Angiopathies" OR "Peripheral Angiopathy" OR "Peripheral Arteriopathies" OR "Peripheral Arteriopathy" OR "Peripheral Vasculopathies" OR "Peripheral Vasculopathy" OR "Monckeberg Medial Calcific Sclerosis" OR "Monckeberg's Medial Calcific Sclerosis" OR "Monckeberg's Sclerosis" OR "Monckeberg Sclerosis" OR "Monckebergs Sclerosis" OR "Mönckeberg's Medial Calcific Sclerosis" OR "Mönckeberg's Sclerosis" OR "Mönckeberg Sclerosis" OR "Mönckebergs Sclerosis" OR "Monckeberg" OR "Monckeberg*" OR "Moenckeberg" OR "Moenckeberg*" OR "Medial Calcific Sclerosis" OR "Medial Calcific Scleroses" OR "Mönckeberg Medial Calcific Sclerosis" OR "incompressible arter*" OR "incompressible vessel*" OR "medial calcified arter*" OR (("Peripheral Occlusive Arterial Disease" OR "Peripheral Vascular Disease") AND ("peripheral" OR "peripheral*" OR "limbs" OR "limb" OR "leg" OR "legs" OR "extremity" OR "extremities" OR "arm" OR "arms")) OR "Intermittent Claudication" OR "Intermittent Claudication" OR "Leriche Syndrome" OR "Leriche Syndrome" OR "Leriche's Syndrome" OR "Leriches Syndrome" OR "Arteriosclerosis Obliterans" OR (("Atherosclerosis" OR "Arteriosclerosis" OR "Atherosclerosis" OR "Arteriosclerosis") AND ("peripheral" OR "peripheral*" OR "limbs" OR "limb" OR "leg" OR "legs" OR "extremity" OR "extremities" OR "arm" OR "arms"))):ti,ab,kw AND ("Ankle Brachial Index" OR "Ankle Brachial Indices" OR "Ankle Brachial Index" OR "Ankle Brachial Indices" OR "anklebrachial index" OR "anklebrachialindex" OR "ankle brachial" OR "anklebrachial" OR "ABI" OR "Toe Brachial Index" OR "Toe Brachial Indices" OR "Toe Brachial Index" OR "Toe Brachial Indices" OR "Toe Pressure" OR "toe brachial" OR "toebrachial" OR "TBI" OR "TP" OR "Oximetry" OR "Oximetry" OR "Oximetries" OR "Oximetr*" OR "Pulse Oximetries" OR "Pulse Oximetry" OR "transcutaneous oxygen tension" OR "transcutaneous oxygen" OR "Pulse" OR "Pulse*" OR "pulsation" OR "pulsations" OR "Transcutaneous Oxygen Monitoring" OR "Transcutaneous Blood Gas Monitoring" OR "Transcutaneous Capnometry" OR "PtcO2" OR "TcPCO2" OR "Doppler waveform" OR "Doppler waveforms" OR "Doppler wave form" OR "Doppler wave forms" OR "pulsed Doppler echocardiography" OR "Pulsed Doppler" OR "Doppler Pulsed" OR "non invasive" OR "non invasiv*" OR "point of care test" OR "point of care tests"):ti AND ("Angiography" OR "Angiography" OR "Angiographies" OR "Angiogram" OR "Angiograms" OR "Angiogra*" OR "Arteriography" OR "Arteriographies" OR "Arteriogra*" OR "Digital Subtraction Angiography" OR "DSA" OR "Magnetic Resonance Angiography" OR "MRI Angiography" OR "MRI Angiographies" OR "Magnetic Resonance Angiography" OR "Magnetic Resonance Angiographies" OR "MRA" OR "perfusion weighted imaging" OR "Perfusion Magnetic Resonance Imaging" OR "Perfusion Weighted MRI" OR "Computed Tomographic Angiography" OR "Computed Tomography Angiography" OR "Computed Tomography Angiographies" OR "CT Angiography" OR "CT Angiographies" OR "CTA" OR "duplex Doppler ultrasonography" OR "Duplex Doppler" OR "Doppler Duplex" OR "color Doppler flowmetry" OR "Doppler Color" OR "Color Doppler" OR "Doppler Colour" OR "Colour Doppler" OR "color duplex" OR "colour duplex" OR "duplex color" OR "duplex colour" OR "CDUS" OR "Plethysmography" OR "Plethysmography" OR "Plethysmogra*" OR "color spectral waveforms" OR "color spectral waveform" OR "color spectral wave forms" OR "color spectral wave form" OR "colour spectral waveforms" OR "colour spectral waveform" OR "colour spectral wave forms" OR "colour spectral wave form"):ti) | 10  0 meeting abstracts | 4 |
|  |  |  |  |
| Emcare | See Embase | 185 | 36 |
|  |  |  |  |
| total |  | 1586  178 meeting abstracts | 1016  164 meeting abstracts |
